# A Precision-Engineered DC-Targeting mRNA-LNP Neoantigen Vaccine Elicits Stronger T Cell Responses and Exhibits Superior Tumor Control

**DOI:** 10.3390/vaccines14030239

**Published:** 2026-03-05

**Authors:** Qi Liu, Yan Liu, Jinwei Li, Si Huang, Zhiying Chen, Jia Li, Tao Wang, Peipei Zhou, Jiandong Huo, Dehua Li

**Affiliations:** 1 State Key Laboratory of Respiratory Disease, National Clinical Research Center for Respiratory Disease, Guangzhou Institute of Respiratory Health, The First Affiliated Hospital of Guangzhou Medical University, Guangzhou 510120, China; liu_qi@gzlab.ac.cn (Q.L.); liu_yan01@gzlab.ac.cn (Y.L.); 2023210030@stu.gzhmu.edu.cn (J.L.); 2023210031@stu.gzhmu.edu.cn (S.H.); 2023210460@stu.gzhmu.edu.cn (Z.C.); jiali@gzhmu.edu.cn (J.L.); 2 Guangzhou National Laboratory, Guangzhou International Bio Island, No. 9 XingDaoHuanBei Road, Guangzhou 510005, China; zhou_peipei@gzlab.ac.cn; 3 GemPharmatech Co., Ltd., Foshan 528225, China; wangtao@gempharmatech.com

**Keywords:** nanobody, dendritic cell, T cell, mRNA-LNP, vaccine

## Abstract

**Background/Objectives**: Messenger RNA (mRNA) vaccine technology has shown great potential in the prevention of infectious diseases and treatment of cancers, but its full potential is limited by non-specific delivery mediated by the current lipid nanoparticle (LNP) platform. **Methods**: Here, we developed a dendritic cell (DC)-targeting LNP incorporated with an ultra-high-affinity CLEC9A-specific nanobody that facilitates enhanced DC uptake but reduced liver accumulation. We assessed the therapeutic efficacy of nanobody-functionalized lipid nanoparticles (Nb-LNPs) in a mouse Lewis lung carcinoma (LLC) model, alongside an evaluation of T cell-mediated immune responses and dendritic cell activation, facilitated by the delivery of mRNA-based neoantigen vaccines. **Results**: Compared with the use of an unfunctionalized LNP, personalized mRNA cancer vaccines encapsulated with this Nb-LNP demonstrated not only superior anti-tumor effects but also a favorable bio-safety profile in a mouse Lewis lung carcinoma model. The mRNA Nb-LNP neoantigen vaccines also induced substantially higher levels of DC maturation and more potent antigen-specific T cell responses, in particular CD4^+^ T cell responses, which are critical for initiation of anti-tumor immunity and immune memory. **Conclusions**: Taken together, these results suggest that precision-engineered LNPs conjugated with a CLEC9A-specific antibody or nanobody could be a promising platform for delivering mRNA vaccines specifically to dendritic cells, improving their prophylactic or therapeutic effects.

## 1. Introduction

Lung cancer remains the leading cause of cancer-related mortality worldwide. Despite advancements in clinical management, the five-year survival rate for patients with advanced non-small-cell lung cancer continues to be less than 20% [1]. While immune checkpoint inhibitors have shown therapeutic benefits for a subset of patients, only about 30% experience positive outcomes, and these treatments can also lead to immune-related side effects [2]. These challenges highlight the urgent need to develop new and more effective tumor immunotherapy approaches.

A cancer vaccine is a promising immunotherapy regimen for the prevention and treatment of cancers. Tumor antigens are divided into tumor-associated antigens (TAAs) and tumor-specific antigens (TSAs) [3]. While TAAs are self-antigens derived from unmutated genes overexpressed in cancer cells but also expressed in normal cells, TSAs are non-self-antigens, also known as neoantigens, resulting from genetic alterations and they are tumor-specific. Despite enormous efforts that have been made to develop cancer vaccines as prophylactic or therapeutic regimens, early cancer vaccines that mainly target shared TAAs, such as gp100, MAGE-12 and NY-ESO-1, which are widely expressed in different tumor types, have shown limited and unsatisfactory clinical outcomes [4]. This may be due to the fact that TAA vaccines have to overcome central and acquired tolerance, thus inducing limited T cell responses [5]. By contrast, neoantigen vaccines do not encounter these challenges and hence can induce robust immune responses. A number of pre-clinical [6,7,8,9,10,11] and clinical studies [12,13,14,15,16] of neoantigen-based therapeutic vaccines have shown encouraging outcomes.

Neoantigen vaccines can be categorized into shared neoantigen vaccines and personalized neoantigen vaccines. Shared neoantigens, which are expressed in many patient tumors, can be prepared as “off-the-shelf” vaccines [17,18]. Examples of shared neoantigens include mutant forms of KRAS, HRAS and NRAS, which can be found in various cancer types, such as lung cancers and pancreatic cancer [19]. Nonetheless, not all cancer patients contain these shared neoantigens, and recent advances in next-generation sequencing technology have made it feasible to identify multiple personalized neoantigens that are unique to one patient. Through in silico prediction of the binding affinity of the neoantigen-derived epitopes to the patient’s HLA molecules as well as measurement of the expression levels of the neoantigens through tumoral RNA sequencing, highly expressed and potentially immunogenic neo-epitopes can be selected to produce peptide [12] or mRNA vaccines [20].

Compared to peptide vaccines, mRNA vaccines possess the advantages of offering rapid production, having self-adjuvating properties and inducing more robust and sustained immune responses [10,21]. However, the fragile mRNA molecules need to be encapsulated in a vehicle to protect them from degradation and facilitate their entry into host cells. Lipid nanoparticles (LNPs) have been the primary technology used for the delivery of mRNA and have successfully entered the clinic [22]. Notably, the Pfizer/BioNTech and Moderna mRNA vaccines proved to be efficacious and saved millions of lives during the COVID-19 pandemic [23]. However, the full potential of mRNA-LNP technology across various diseases is constrained by its liver-specific tropism [24]. For cancer vaccines, the uptake of cancer vaccines by dendritic cells (DCs) is crucial to the presentation of the neo-epitopes and activation of tumor-specific T cells [25,26]. To enhance cellular uptake of the LNP by DCs, a mannose ligand has been incorporated into the LNP [27,28] to facilitate engagement of the mannose receptor (CD206, MR) on the DC surface and subsequent internalization. However, the mannose receptor is not only expressed on DCs but also on macrophages and endothelial cells [29]. C-type lectin receptor family 9 member A (CLEC9A) is a C-type lectin receptor primarily found on type 1 conventional dendritic cells (cDC1s) and plays a key role in endocytosis. It facilitates the uptake of antigens and their cross-presentation to CD8^+^ T cells [30]. Importantly, the antigen–CLEC9A complex bypasses lysosomal compartments, which enhances antigen preservation and improves cross-presentation efficiency, thereby boosting the effectiveness of cancer immunotherapy [31,32]. As a result, selectively activating cDC1 using CLEC9A-targeted nanovaccines offers a promising approach for cancer immunotherapy research.

Here, we developed a nanobody-functionalized lipid nanoparticle (Nb-LNP) system designed to mediate DC-targeting delivery of mRNA cancer vaccines. This precision-engineered LNP was incorporated with a nanobody with a low-picomolar binding affinity to C-type lectin receptor family 9 member A (CLEC9A), a receptor that is exclusively expressed on type 1 conventional dendritic cells (cDC1s) [33]. This Nb-LNP significantly enhanced delivery of mRNA neoantigen vaccines to DCs in the spleen and lymph nodes and was able to facilitate potent cellular immune responses, in particular CD4^+^ T cell responses, and anti-tumor effects. Our study therefore provides proof of principle that conjugating LNPs with a CLEC9A-specific antibody/nanobody represents a promising approach for enhancing the effects of mRNA cancer vaccines.

## 2. Materials and Methods

### 2.1. Cell Cultures and Mice

Lewis lung carcinoma (LLC) murine cell line was purchased from Bohui Biological Technology (Guangzhou) Co., Ltd. (Guangzhou, China, JCRB, CAT.BH-C419, RRID: CVCL_4358, 2024). Human embryonic kidney epithelial 293T cell line was purchased from ATCC (CAT.CBP60439, RRID: CVCL_0063, Manassas, VA, USA). LLC and HEK-293T cell lines were maintained in Dulbecco’s Modified Eagle’s Medium (DMEM; Gibco, Waltham, MA, USA) supplemented with 10% fetal bovine serum (FBS) [34]. Cultures were incubated at 37 °C in a humidified environment containing 5% carbon dioxide (CO_2_). The Freestyle 293F cell line (Gibco, CAT.R79007, RRID: CVCL_D603) was cultured in Freestyle 293 expression medium under conditions of 37 °C, 8% CO_2_, and continuous agitation at 120 rpm within a humidified atmosphere. All cell lines utilized in this study were confirmed to be free of contamination. Female C57BL/6 mice, aged 6 to 8 weeks, were obtained from GemPharmatech Co., Ltd (Foshan, China). They were kept in a temperature-regulated environment, with unlimited access to food and sterile water, all within a specific pathogen-free setting.

### 2.2. Extraction of Tissue-Derived DNA and RNA for Sequencing

Genomic DNA was extracted from a normal lung of a C57BL/6 mouse. Additionally, tumoral genomic DNA and total RNA were isolated from cultured LLC cell-induced subcutaneous (S.C.) and orthotopic (Ortho) lung tumors, respectively, utilizing the DNeasy Blood & Tissue Kit (CAT.69504, QIAGEN, Hilden, Germany) and TRIzol Reagent (CAT.15596018, Thermo Fisher Scientific, Waltham, MA, USA). To establish an orthotopic lung tumor model, 2 × 10^6^ cells suspended in 20 µL of Matrigel were administered via injection into the left lateral lobe of the lung following a midline laparotomy in C57BL/6 mice. After a period of two weeks, the lung tumors were excised for further analysis. For the induction of a subcutaneous tumor model, 1 × 10^6^ LLC cells suspended in 100 µL of normal saline were administered via subcutaneous injection into the right flank of C57BL/6 mice. The induced tumor was carefully dissected after a period of two weeks.

### 2.3. Next-Generation Sequencing

Whole-exome sequencing (WES) and RNA sequencing (RNA-seq) were conducted on samples from C57BL/6 mice. Genomic DNA was extracted from normal lung tissue and LLC cell-induced tumors for WES, while total RNA was isolated from tumor-bearing lung tissue for RNA-seq. For WES, libraries were prepared using the Agilent (Santa Clara, CA, USA) SSELXT Mouse All Exon V6 Kit. Genomic DNA was fragmented to ~300 bp using hydrodynamic shearing, followed by end repair, 3′ adenylation and adapter ligation. Fragments possessing adapters at both termini underwent PCR amplification and were subsequently hybridized with biotin-labeled probes specific to exonic regions. Captured fragments were isolated using streptavidin-coated magnetic beads, further amplified by PCR, and indexed. RNA sequencing libraries were constructed using the VAHTS^®^ Universal V8 RNA-seq Library Preparation Kit (CAT.NR605-01, Vazyme, Nanjing, China). Polyadenylated mRNA was isolated from total RNA through the use of poly-T oligo-conjugated magnetic beads and subsequently fragmented by thermal treatment. First-strand cDNA synthesis was conducted by employing random primers, followed by second-strand synthesis in which deoxyuridine triphosphate (dUTP) was incorporated in the place of deoxythymidine triphosphate (dTTP) [35]. Subsequent to end repair and 3′ adenylation, sequencing adapters containing index sequences were ligated to the cDNA fragments. Polymerase chain reaction (PCR) amplification was then performed utilizing a 2× PCR Master Mix. The resulting libraries were purified using the AMPure (Brea, CA, USA) XP system and quantified via the Agilent High Sensitivity DNA assay. Sequencing was performed using the Illumina (San Diego, CA, USA) NovaSeq 6000 platform, generating paired-end reads with a length of 150 base pairs.

### 2.4. Bioinformatic Analyses

The quality of raw reads from whole-exome sequencing (WES) data was evaluated using FastQC (version 0.11.7). Subsequently, adaptor sequences were trimmed by employing Fastp (version 0.23.4). Following quality control procedures, the processed reads were aligned to the mouse reference genome (GRCm38) utilizing the Burrows-Wheeler Aligner (BWA, version 0.7.17). Samtools (v1.5) was used for alignments in the SAM format and sorting them into BAM files. In the alignment data, duplicated reads were flagged by the Genome Analysis Toolkit (GATK, v4.0.5.1), and base quality scores were also recalibrated using the GATK [36]. The output file was used as input for Samtools to build the sequence index. Using the existing mouse SNP/INDELS database, the BaseRecalibrator function embedded in the GATK tool was used to establish a correlation model and generate a recalibration table, and the ApplyBQSR function of the GATK tool was used to adjust the original bases. The somatic variant calling was conducted utilizing the Mutect2 tool within the GATK framework, comparing normal lung tissue to LLC cell-induced tumors in C57BL/6 mice. The Variant Call Format (VCF) files generated were filtered based on the “PASS” criterion. Subsequent annotation of somatic VCF files was performed using ANNOVAR (version 2020-06-07). The variant allele frequency (VAF) was calculated by dividing the read depth of the mutated allele by the overall sequencing depth within tumor samples. Variants exhibiting a VAF greater than 10% were classified as true positives. Non-synonymous mutations were then selected according to the annotation results. For RNA sequencing data, the quality of raw reads was assessed using FastQC, and adaptor sequences were removed by employing Cutadapt (version 4.4). Following quality control, sequencing reads were aligned to the mouse reference genome GRCm38 using the HISAT2 alignment tool (version 2.2.1). Read counts were obtained using featureCounts (version 2.0.6), and gene expression levels were normalized to fragments per kilobase of transcript per million mapped reads (FPKM).

### 2.5. Prediction, Selection and Prioritization of Neoantigens

Among the identified non-synonymous mutations, those exhibiting a variant allele frequency (VAF) of at least 0.1 and a fragments per kilobase of transcript per million mapped reads (FPKM) value of at least 1 were subsequently selected for in silico assessment of their binding affinity to MHC class I and class II molecules [36]. This prediction was conducted using netMHCpan-4.0a and netMHCIIpan-4.0, respectively. (IEDB, http://tools.iedb.org/mhci/, accessed on 5 March 2025). Specifically, we translated the nucleic acid sequence into an amino acid sequence and selected peptides containing the mutation site with a length of 8–10 amino acids as the input for the prediction of binding affinity (BA) with the MHC-I molecules and peptides with a length of 12–14 amino acids for the MHC-II molecules. Since the haplotype of C57BL/6 mice is H-2b, we choose to predict the BA with H2-Kb and H2-Db MHC-I molecules and with I-Ab MHC-II molecules. The antigen peptide prediction results were sorted in ascending order according to the BA score, and high-affinity peptides were selected as core candidate peptides. Longer peptides offer distinct advantages compared to shorter peptides, such as enhanced presentation on MHC molecules and the capacity to activate both CD8^+^ and CD4^+^ T cells. Consequently, left and right extensions were conducted on the predicted core peptides (encompassing both MHC-I and MHC-II), resulting in peptides of 27 amino acids in length, with the mutation site positioned at residue 14. Ultimately, ten MHC-I and ten MHC-II peptide sequences exhibiting the highest binding affinities were selected for synthesis and utilized in subsequent experimental procedures.

### 2.6. Peptides and Antibodies

Each neoantigen candidate was chemically synthesized as a peptide by QYAOBIO (Shanghai, China). The peptides were produced with lengths ranging from 8 to 14 amino acid residues. V500 Rat Anti-Mouse CD45 (CAT.561487), Fixable Viability Stain 700 (CAT.564997), APC-Cy7 Hamster Anti-Mouse CD3e (CAT.557596), RB705 Anti-Mouse CD4 GK1.5 (CAT.570257), APC Rat Anti-Mouse CD45R/B220 (CAT.553092), BV421 Rat Anti-Mouse F4/80 (CAT.565411), APC Hamster anti-Mouse CD80 (CAT.560016), PE-Cy7 Rat Anti-Mouse CD86 (CAT.560582), PE Rat Anti-Mouse I-A/I-E (CAT.557000), FITC Rat Anti-Mouse CD11b (CAT.557396), RB705 Anti-Mouse CD11c (CAT.570627), BB515 Anti-Human IgG (CAT.564581), Rat Anti-Mouse CD16/CD32 (CAT.553142) were purchased from BD Pharmingen (Franklin Lakes, NJ, USA). Alexa Fluor^®^ 488 Anti-mouse CD8a (CAT.100723) was purchased from Biolegend (San Diego, CA, USA). PE Anti-Mouse CD370 (CAT.12-5975-82), APC Anti-mouse IFN γ XMG1.2 (CAT.17-7311-82), and PE Anti-mouse TNF α MP6-XT22 (CAT.12-7321-82) were purchased from Invitrogen (Carlsbad, CA, USA).

### 2.7. Protein Production

All genes utilized in this study were synthesized by GenScript (Nanjing, China) and codon-optimized for expression in human cells [34]. To mitigate the risk of autoimmune responses, the Immunoglobulin G (IgG1) fragment crystallizable (Fc) sequence employed in this research was designed in accordance with previously published methodologies. Briefly, to construct the Mus musculus C-type lectin domain family 9 member A (mCLEC9A) extracellular domain (ECD), Fc, the mCLEC9A ECD sequence (PDB#3J82), was fused to the N-terminus of the IgG1 Fc region using a (G4S)_2_ linker. To produce the secreted protein, an IL-2 signal peptide was fused to the N-terminus of the mCLEC9A extracellular domain (ECD). The expression vectors were temporarily introduced into Freestyle 293F cells using polyethyleneimine (PEI) as the transfection agent. Seven days post-transfection, the culture supernatants were harvested and subjected to centrifugation to eliminate cellular debris. Subsequently, the recombinant protein was purified via affinity chromatography utilizing nickel–nitrilotriacetic acid (NTA) resin under physiological pH conditions (pH 7.4) (CAT.17371202, Cytiva, Washington, WA, USA). pcDNA3.1-mCLEC9A-AviTag was constructed to express the monomeric form of the mCLEC9A protein. The Avi-Tag sequence was appended to the C-terminus of the protein to facilitate intracellular biotinylation. This vector was co-transfected with a plasmid encoding the biotin ligase enzyme (BirA) into Freestyle 293F cells. Biotin substrate was supplemented in the culture medium to enable enzymatic biotinylation. After a 5-day incubation period, the protein-containing supernatant was collected and the target protein was purified via its His-tag. The extent of biotinylation was subsequently assessed using an ELISA assay. The pADL-23c vector utilized for nanobody expression contains a PelB leader sequence and a C-terminal hexahistidine (6× His) tag. The plasmids were transformed into the WK6 strain of *Escherichia coli*, and protein expression was initiated by adding 1 mM IPTG, followed by incubation overnight at 20 °C. Periplasmic extracts were obtained via osmotic shock, and VHH proteins were subsequently purified through immobilized metal affinity chromatography (IMAC) using an automated protocol on an AKTXpress system [37]. This purification was further refined by size-exclusion chromatography on a Superdex 75 10/300GL column, employing DPBS at pH 7.4 as the buffer. In parallel, 2A4-Fc proteins were generated by transient transfection of Freestyle 293F cells and purified using AmMag™ Protein A Magnetic Beads (GenScript, Nanjing, China), followed by gel filtration chromatography in DPBS at pH 7.4.

### 2.8. Llama Immunization and Construction of Nanobody Library

A llama was administered biweekly immunizations using a combination of mCLEC9A ECD-Fc fusion protein and Gerbu LQ adjuvant. Following the completion of three immunization sessions, a volume of 100 mL of camel blood was obtained for the purpose of isolating peripheral blood mononuclear cells (PBMCs). Subsequently, total RNA was extracted from these cells, followed by complementary DNA (cDNA) synthesis. Two successive rounds of PCR were then performed to amplify the variable domain of the heavy chain of heavy-chain antibody (VHH) fragments. The VHH cDNAs were purified by agarose gel electrophoresis and then cloned into the SfiI sites of the phagemid vector pADL-23c. A pelB leader sequence precedes the VHH encoding sequence in this vector, which is then followed by a 6× His and cMyc tag. Electrocompetent *Escherichia coli* TG1 cells were transformed with the recombinant pADL-23c vector, resulting in a VHH library comprising independent transformants. Subsequently, the TG1 library stock was infected with M13K07 helper phage to generate a library of phages displaying VHH fragments [37].

### 2.9. Isolation of Nanobodies

Phages presenting VHHs specific to the extracellular domain of mCLEC9A were selectively enriched following two successive rounds of bio-panning [38,39]. These rounds were conducted using 50 nM and 5 nM concentrations of biotinylated mCLEC9A ECD, respectively, with capture facilitated by Dynabeads™ M-280 (CAT.11205D, Thermo Fisher Scientific, Waltham, MA, USA). The extent of enrichment after each panning cycle was evaluated by plating the cell culture through tenfold serial dilutions. Subsequent to the second panning round, a total of 94 individual phagemid clones were isolated. Phages displaying VHH were then rescued through infection with the M13K07 helper phage and subsequently evaluated for their binding affinity to the mCLEC9A ECD via ELISA [38]. In these experiments, the extracellular domain of mCLEC9A was immobilized onto a 96-well microplate, after which the binding interactions of phage clones were evaluated within the plate. The phage particles that remained bound were detected using a horseradish peroxidase-conjugated anti-M13 antibody (CAT.11973-MM05T-H, Sino Biological, Beijing, China; 1:5000) [37].

### 2.10. Biolayer Interferometry (BLI)

The ForteBio Octet system (ForteBio, Santa Clara, CA, USA) was employed to evaluate the binding affinity of highly potent VHHs to the mCLEC9A ECD antigen. Streptavidin biosensors were initially equilibrated in PBST buffer for a duration of 10 min. Following this, the biosensors were incubated with diluted biotinylated mCLEC9A ECD protein. After thorough mixing, the biosensors were subsequently immersed in PBST buffer. Thereafter, the biosensors underwent association assays with a series of diluted, highly potent VHH samples. The kinetic parameters, including association rate constants (*K*on), dissociation rate constants (*K*off), and equilibrium dissociation constants (*K*_D_), were determined using ForteBio Data Analysis software version 12.0 [40].

### 2.11. mRNA Construct and Synthesis

The neoantigen sequences were inserted into the mRNA platform backbone. Furthermore, synthetic DNA fragments encoding five putative neo-epitopes—each comprising 27 amino acids with the mutation positioned at residue 14—were constructed [6]. These epitopes were linked via non-immunogenic glycine/serine linkers, specifically a central linker sequence of GGSGGGGSGG and terminal linkers of GGSLGGGGSG. The assembled sequences were cloned into an initial vector containing signal peptide (SP) and major histocompatibility complex class I (MHCI) trafficking signal (MITD) domains (SP sequence: MRVTAPRTLILLLSGALALTETWAGS; MITD sequence: IVGIVAGLAVLAVVVIGAVVATVMCRRKSSGGKGGSYSQAASSDSAQGSDVSLTA) designed to optimize trafficking through MHC class I and II antigen presentation pathways. Additionally, the vector incorporated backbone elements intended to enhance RNA stability and translational efficiency [6]. The plasmid deoxyribonucleic acid was purified using PureLink™ HiPure Plasmid DNA Purification Kits (CAT.K210004, Thermo Fisher Scientific, Waltham, MA, USA). The preparation was conducted through a linearization procedure employing the XhoI endonuclease (CAT.R0146S, New England Biolabs, Ipswich, MA, USA). The in vitro transcription technique was employed to synthesize mRNA sequences encoding luciferase, eGFP, and neoantigens (Figure S5C). The T7 High Yield RNA Synthesis Kit (AGCN) (CAT.10110N, Jiangsu Synthgene Biotechnology, Jiangsu, China) was employed to synthesize RNA from DNA templates following the manufacturer’s protocol. Subsequently, DNase I was introduced into the transcription mixture and incubated at 37 °C for 30 min to degrade the DNA template [41]. The resultant mRNA underwent additional purification via lithium chloride (LiCl) precipitation. RNA integrity and quality were assessed by agarose gel electrophoresis, and the final RNA concentration was determined using a NanoDrop microdevice (Thermo Fisher Scientific, Waltham, MA, USA) [41].

### 2.12. LNP Formulation and Characterization

SM-102, distearoylphosphatidylcholine (DSPC), polyethylene glycol (DMG-PEG2000), DSPE–PEG (2000)–maleimide, and cholesterol for LNP formulation with mRNA were purchased from AVT (Shanghai, China) Pharmaceutical Tech Co., Ltd. All lipid components were dissolved in ethanol for long-term storage. The mRNA was solubilized in a citrate buffer with a pH of 5.0. Thereafter, the lipid mixture and mRNA were combined at a volumetric ratio of 1:3 utilizing a microfluidic device, specifically the INano L^+^ system (Micro & Nano, Shanghai, China). The LNP-mRNA formulations were diluted 10-fold in 1× DPBS buffer (pH 7.2–7.4) and concentrated by ultrafiltration with the Amicon Ultra Centrifugal Filter Unit (Merck, Darmstadt, Germany) [41]. The RNA concentration and encapsulation efficiency were measured using the Quant-it RiboGreen RNA Assay Kit (CAT.R11490, Invitrogen, Carlsbad, CA, USA) [42].

### 2.13. Nb-LNP Conjugation

Anti-mCLEC9A nanobody was conjugated to 1,2-distearoyl-sn-glycer o-3-phosphoethanolamine (DSPE)–PEG–maleimide via a C-terminal cysteine post-insertion technique. The proportion of all lipid components was a molar ratio of 50:10:38.5:1.25:0.25 for Ionizable cationic lipid:DSPC:cholesterol:DMG-PEG2000:DSPE–PEG–maleimide [43]. The mCLEC9A nanobody 2A4 was subjected to reduction using Tris(2-carboxyethyl) phosphine hydrochloride (TCEP) (CAT.ST045, Beyotime, Shanghai, China) through incubation for 60 min at room temperature. Subsequently, TCEP was removed by centrifugation employing a 3 kDa molecular weight cutoff filter (Millipore Sigma, Darmstadt, Germany), and the antibody was resuspended in 100 μL of DPBS. Maleimide-functionalized lipid nanoparticles (Mal-LNPs) were then conjugated with the reduced antibody at a molar ratio of 1:1 (maleimide to antibody). Following 1 h incubation at room temperature, the mixture was maintained at 4 °C overnight to ensure completion of the conjugation reaction, and unbound nanobodies were removed via filtration through a 100 kDa molecular weight cutoff centrifugal filter unit [44]. The sizes, distributions and zeta potential of mRNA-LNPs were measured using the Malvern Zetasizer Nano ZS dynamic light scattering instrument (Malvern Instruments, Malvern, UK) [45].

### 2.14. Enzyme-Linked ImmunoSpot (ELISpot)

Female C57BL/6 mice aged 6 to 8 weeks were administered 10 µg of mRNA-LNP vaccine on days 0, 14, and 28. Seven days after the administration of the final vaccine dose, the mice were euthanized, and splenocytes were harvested for the evaluation of immunogenicity using ELISpot and flow cytometry assays. Splenocytes from immunized mice (2–5 × 10^5^ cells) were cultured for 48 h in anti-mouse IFN-γ mAb (AN18) (CAT.3321-3-1000, Mabtech)-coated 96-well plates. The quantity of spots was quantified utilizing an automated ELISPOT reader.

### 2.15. Generation of Constructs and Transduced Cell Lines

The full-length open reading frame (ORF) of mCLEC9A was inserted into the pLVX-EF1α-IRES-Puro/ZsGreen (YouBio, Chongqing, China) retroviral vector by GenScript. Lentiviral particles were produced by transfecting HEK-293T cells with the plasmid DNA in combination with the packaging vectors psPAX2 and pMD2.G, using a molar ratio of 4:3:1. The transfection procedure was facilitated using the JetPRIME in vitro DNA Transfection Reagent (CAT.101000046 (114-15), Polyplus, Strasbourg, France). The culture medium was replaced after 6 h. Subsequently, 48 h following the medium change, the virus-containing medium was collected and concentrated. Viral supernatants were subjected to centrifugation at 1500× *g* for a duration of 45 min, after which the resultant viral pellets were resuspended in DMEM medium. The lentiviral preparations were subsequently stored at −80 °C for later application. For the transduction procedure, 1 × 10^5^ HEK-293T cells were plated in each well of 6-well culture plates, concurrently supplemented with 400 μL of lentivirus and 8 μg/mL polybrene (CAT.28728-55-4, Selleck, Houston, TX, USA). Twenty-four hours post-passage, lentivirus-infected cells were subjected to selection with puromycin (CAT.P8833, Sigma-Aldrich, Saint Louis, MO, USA) at a concentration of 1 μg/mL for a duration of 3 days [46]. The binding of 2A4-Fc to mCELC9A^+^HEK-293T cells was validated via flow cytometry analysis. Nanobodies (10 μg/mL) were introduced to the cells suspended in FACS buffer and incubated for 30 min. Following three washing steps, the cells were subsequently incubated with the secondary antibody for an additional 30 min. After washing thrice in FACS buffer, binding was detected by flow cytometry.

### 2.16. Generation of FL-DCs from Bone Marrow

Flt3L-Bone Marrow-Derived Dendritic Cells (FL-DCs) expressing CLEC9A were induced by culturing bone marrow cells flushed from the femurs of 6–8-week-old C57BL/6 mice [47]. The culture medium was supplemented with 50 ng/mL Flt3 ligand (Flt3L, CAT.CC19-50, NOVOPROTEIN, Suzhou, China) and 1.5 ng/mL granulocyte–macrophage colony-stimulating factor (GM-CSF, CAT.CK02-10, NOVOPROTEIN, Suzhou, China). Half of the medium was replaced every 4 or 5 days. On day 15, non-adherent and loosely adherent immature DCs were harvested. Subsequently, 1 × 10^6^ FL-DCs and mCLEC9A-positive HEK-293T cells were harvested for flow cytometric analysis. The cell suspension was incubated with anti-CD16/32 antibody at 4 °C for 10 min to block Fc receptors, followed by staining with PE-conjugated Anti-Mouse CD370 antibody at 4 °C for 30 min. After two washes, the cells were subjected to flow cytometric evaluation.

### 2.17. In Vitro Luciferase and Toxicity Assays

mCLEC9A^+^ HEK-293T cells and FL-DCs were seeded at a density of 3 × 10^4^ cells per 100 μL of supplemented DMEM or RPMI-1640 medium (containing 10% FBS and 1% penicillin–streptomycin) in 96-well plates before being treated with lipid nanoparticles. To assess the delivery of luciferase mRNA at 24 h or at different time intervals, the cells were resuspended in 80 μL of lysis buffer (0.1% TritonX100) and 80 μL of luciferase assay substrate (CAT.DD1204-03, Vazyme, Nanjing, China). Luminescent signals were quantified using the BioTek (Winooski, VT, USA) Synergy Neo plate reader. mCLEC9A^+^ HEK-293T cells were plated into 96-well plates at a density corresponding to 100 μL of culture medium per well and subsequently incubated for a duration of 24 h. Subsequently, the cells were treated with mRNA-loaded Mal-LNP or Nb-LNP formulations across a range of mRNA concentrations for an additional 24 h. At the time of measurement, the plates were equilibrated to room temperature, followed by the addition of 100 μL of CellTiter-Glo reagent (CAT.G7572, Promega, Madison, WI, USA) to each well. The plate contents were mixed for 2 min on an orbital shaker to facilitate cell lysis and then incubated at room temperature for 10 min to allow for stabilization of the luminescent signal [45]. Luminescence was then measured using a microplate reader.

### 2.18. Bioluminescence and Fluorescence Imaging

For in vivo imaging studies, C57BL/6 mice received intramuscular (i.m.) injections of LNP formulations encapsulating mRNA encoding luciferase (Figure S5C). Each mouse was administered 50 μL of DPBS containing 5 μg of mRNA luciferase into a single quadriceps muscle, located in the anterior thigh region. For ex vivo organ imaging, animals were intraperitoneally injected with D-luciferin dissolved in PBS at a concentration of 15 mg/mL, administered five minutes prior to euthanasia [48]. Subsequently, the organs were meticulously harvested and maintained under dark conditions to preserve luminescent signal integrity. Subsequently, the organs were rinsed three times with DPBS to eliminate residual blood from their surfaces. Bioluminescence imaging was then conducted utilizing the IVIS Lumina system. Luminescence intensity was quantified within predefined regions of interest (ROIs) to assess the distribution and expression efficiency of the mRNA-Luc-LNP [48].

### 2.19. eGFP mRNA Expression in Different Cell Types and DC Maturation

To assess mRNA expression across various cell types within lymph nodes and spleen, eGFP mRNA encapsulated in LNPs was administered intramuscularly at a dose of 10 μg mRNA (Figure S5C), 36 h prior to analysis. Subsequently, spleen and inguinal lymph nodes were harvested and mechanically dissociated using 70 µm cell strainers to generate single-cell suspensions. Erythrocytes were eliminated using red blood cell lysis buffer, and the resulting cells were collected, washed, and incubated with anti-mouse CD16/32 antibody at 4 °C for 10 min to block Fc receptors [49]. Flow cytometric analysis was then performed to quantify GFP-positive cells among distinct cell populations, following staining with antibodies specific for T cells, B cells, dendritic cells (DCs), and macrophages. For evaluation of DC maturation, C57BL/6 mice received a single intramuscular injection of Mal-LNP or Nb-LNP formulations containing 10 μg mRNA. Cell surface marker expression was assessed by staining with RB705 anti-mouse CD11c, PE Rat anti-mouse I-A/I-E, APC Hamster anti-mouse CD80, and PE-Cy7 Rat anti-mouse CD86 antibodies. The cells that had been stained were subsequently analyzed using flow cytometry.

### 2.20. Mouse Tumor Model

C57BL/6 female mice purchased from GemPharmatech (Foshan, China) were subcutaneously inoculated with LLC cells at a density of 8 × 10^5^ per mouse. The animals were housed in a specific pathogen-free facility maintaining air humidity levels between 40% and 70%, an ambient temperature of 22 ± 1 °C, and a 12 h light–dark cycle [48]. Following inoculation, the mice were provided with standard feeding for a period of 10 days prior to vaccine administration. Tumor-bearing mice received intramuscular injections of mRNA-LNP on days 13 and 20 post-inoculation. Tumor sizes were measured using an electronic vernier caliper at 3-day intervals, and tumor volume was calculated using the equation V (mm^3^) = (length × width^2^) ÷ 2 [50]. In accordance with established animal welfare guidelines, mice were euthanized upon experiencing a reduction of 20% or more in their initial body weight or when tumor volume surpassed 2000 mm^3^.

### 2.21. Intracellular Cytokine Staining

A total of 2 × 10^6^ cells isolated from spleen or lymph nodes were resuspended in 200 μL of RPMI-1640 culture medium enriched with 10% FBS. To inhibit intracellular cytokine trafficking, GolgiPlug protein transport inhibitor (CAT.555029, BD Biosciences, Franklin Lakes, NJ, USA) was added. For the evaluation of antigen-specific cytokine secretion, the cells were stimulated with 1–2 μg per well of MHC class I and class II peptides and incubated at 37 °C in a humidified environment containing 5% carbon dioxide (CO_2_) for 12 h. Following stimulation, cells were stained according to the established immune cell population analysis protocol. Subsequently, cells were permeabilized using Cytofix/Cytoperm solution (CAT.554714, BD Biosciences) and incubated with fluorescence-conjugated mouse anti-IFN-γ and anti-TNF-α antibodies for 30 min at 4 °C. Following two further washing procedures, the cells were subjected to analysis utilizing an Agilent Novocyte Advanteon Flow Cytometer.

### 2.22. Safety Evaluation

The acute toxicity of intramuscularly administered 10 μg mRNA-LNP complexes was assessed in C57BL/6 mice aged 6 to 8 weeks [51]. Following a 24 h incubation period, whole blood samples were obtained and subjected to centrifugation at 5000 revolutions per minute for 15 min to separate the serum. Biochemical assays evaluating organ function, including measurements of albumin (ALB), alanine aminotransferase (ALT), aspartate aminotransferase (AST), alkaline phosphatase (ALP), total protein (TP), UREA, and creatinine (CREA), were conducted using an automated analyzer (Chemray800, Rayto, Shenzhen, China) [51]. Furthermore, major organs, specifically the heart, liver, spleen, lung, and kidney, were harvested for histopathological examination via hematoxylin and eosin (H&E) staining (Servicebio, Wuhan, China) [52].

### 2.23. Quantification and Statistical Analysis

Statistical significance was determined at a threshold of *p* < 0.05 using Prism Version 9.0 software (GraphPad, San Diego, CA, USA)). Exact *p*-values are provided adjacent to the corresponding lines in the relevant figures, with asterisks indicating the level of significance as follows: * for 0.01 < *p* < 0.05, ** for 0.001 < *p* < 0.01, *** for 0.0001 < *p* < 0.001, and **** for *p* < 0.0001. Information pertaining to the statistical analyses conducted, sample sizes (*n*), median values, and the groups compared is provided in the figure legends.

### 2.24. Ethics Statement

All experimental procedures involving animals were examined and authorized by the Institutional Animal Care and Use Committee at Guangzhou National Laboratory (approval number GZLAB-AUCP-2025-01-A02). The animals were maintained in a temperature-regulated environment and subjected to a 12 h light/dark cycle. They were provided with free access to food and sterile water and housed under specific pathogen-free conditions.

## 3. Results

### 3.1. Isolation and Binding Characterization of Nanobodies That Target mCLEC9A

**Figure 1:**
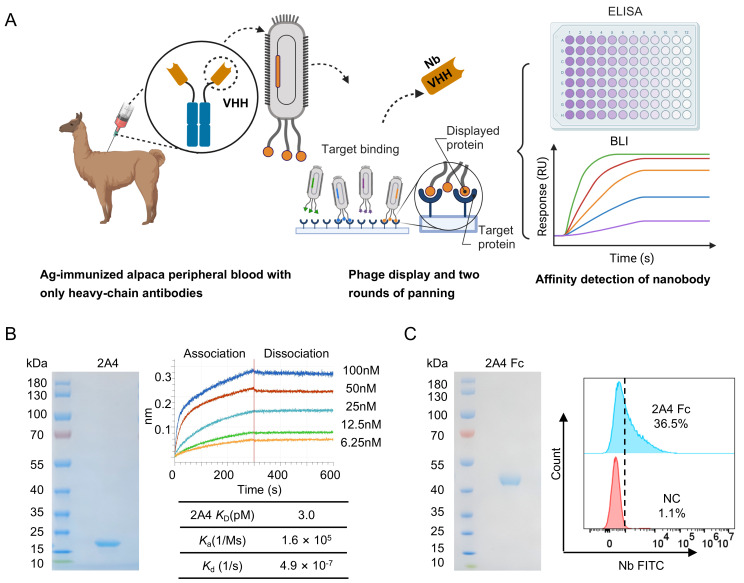
Isolation and characterization of mCLEC9A-specific nanobodies. (**A**) The workflow for the isolation and characterization of nanobodies. (**B**) SDS-PAGE/Coomassie blue staining of purified anti-mCLEC9A nanobody 2A4. Measurement of the affinity of 2A4 to mCLEC9A by Biolayer Interferometry (BLI). Two-fold serial dilutions of 2A4 from 100 nM to 6.25 nM were used to measure binding to biotinylated mCLEC9A immobilized on the Octet BLI biosensor. The equilibrium dissociation constant (*K*_D_) and binding kinetic parameters are presented. (**C**) SDS-PAGE/Coomassie blue staining of purified 2A4-Fc and flow cytometry histograms showing binding of 2A4-Fc to mCLEC9A^+^ HEK-293T cell line. Uncropped SDS-PAGE images can be found in Supplementary File S1.

The workflow of nanobody isolation and characterization is outlined in Figure 1A. Nanobodies targeting mCLEC9A were raised in a llama sequentially immunized three times with mCLEC9A fused to the Fc fragment of human IgG1 (Figure S1A). A phage-displayed nanobody library was constructed from the cDNA of peripheral blood mononuclear cells (PBMCs) of the immunized llama. mCLEC9A binders were selected through two rounds of bio-panning, and unique clones were classified by sequencing (Figure S1B). Five nanobodies were identified and selected for production (Figure 1B and Figure S1C). Their binding kinetics to mCLEC9A were determined, with the best binder 2A4 showed a remarkably high binding affinity with a *K_D_* of 3.0 pM (Figure 1B and Figure S1D). Binding of 2A4-Fc (fusion of the nanobody to the Fc fragment of human IgG1) to mCLEC9A-expressing (mCLEC9A^+^) HEK-293T was verified by flow cytometry (Figure 1C).

### 3.2. Engineered Nb-LNP Exhibits Efficacious Targeted Delivery

**Figure 2:**
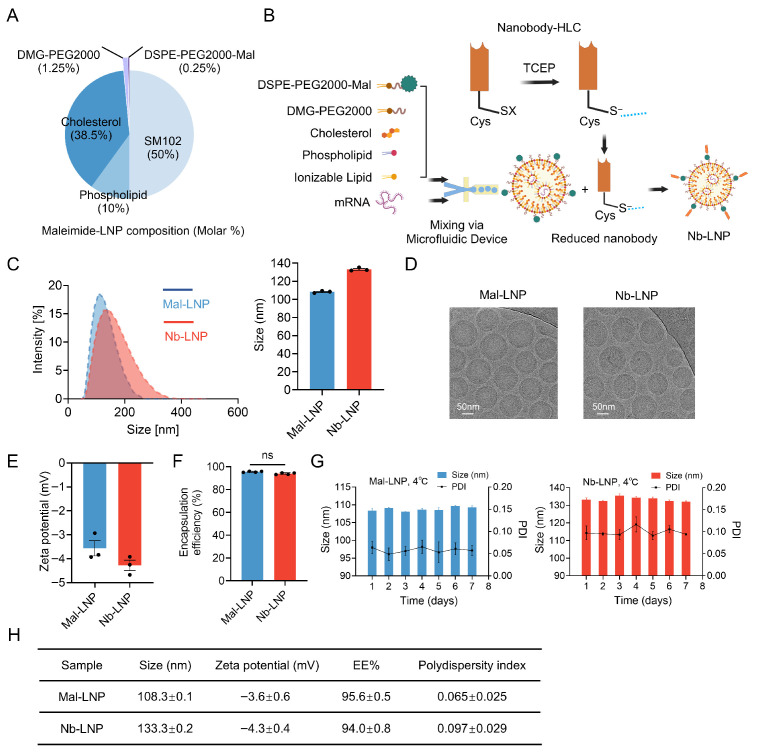
Preparation and characterization of mRNA-LNP complexes. (**A**) Molar composition of maleimide–LNP (Mal-LNP). (**B**) Diagram showing preparation of mRNA-LNP and formation of Nb-LNP through chemical conjugation. (**C**) Measurement of size distribution of Nb-LNP and Mal-LNP by DLS. (**D**) Cryo-EM images of Mal-LNP and Nb-LNP. Scale bar indicates 50 nm. (**E**) Measurement of zeta potential of Nb-LNP and Mal-LNP by DLS. (**F**) Measurement of mRNA encapsulation efficiency for Nb-LNP and Mal-LNP with the use of the Quant-it RiboGreen RNA Assay Kit. (**G**) In vitro stability of Mal-LNP and Nb-LNP at 4 °C for 7 days. (**H**) The measured values for encapsulation efficiency, zeta potential and mean size. All measurements were made with triplicates at pH 7. Statistical analyses were performed using two-way ANOVA. All results are presented as mean ± SEM; “ns”, no significant difference.

To generate nanobody-functionalized LNPs (Nb-LNPs), maleimide-functionalized lipid-anchored PEG (Mal-PEG) was included in the LNP formulation as previously described (Figure 2A) [45]. Nanobody 2A4 was added with a hexahistidine-hIgA1 hinge linker–cysteine (HLC) at its C-terminus (Figure S2A) to allow for conjugation of tris (2-carboxyethyl) phosphine (TCEP)-reduced nanobody to the surface of LNP through a “click chemistry” reaction with maleimide–LNP (Mal-LNP) (Figure 2B and Figure S2B,C). Maleimide and thiol were conjugated at a 1:1 molar ratio, and the C-terminal cysteine of the nanobodies were unable to anchor all the maleimide on the LNP spherical surface due to steric hindrance [53]. To determine the efficiency of nanobody capture onto the LNPs, we used native polyacrylamide gel electrophoresis followed by Western blot to detect the nanobody to measure the amount of free nanobody in solution after functionalization. There was minimal detectable free nanobody for nanobody:maleimide ratios of 1:64, 1:32, 1:16 and 1:8 (Figure S2D), indicating that the nanobody capture was quantitative and that the maximum molar ratio of nanobody conjugated to maleimide groups on the Mal-LNP surface was approximately 1:8 (molar ratio).

The physicochemical characteristics of the nanoparticles were evaluated by employing a size analyzer and cryo-electron microscopy (Cryo-EM). These techniques yielded quantitative data regarding particle size, polydispersity index, surface charge, and morphological features. The results indicated that the particle size of Mal-LNP (108.3 ± 0.1 nm) is smaller than that of Nb-LNP (133.3 ± 0.2 nm), which increased by nearly 25 nm after nanobody modification, and both formulations exhibit a spherical structure (Figure 2C,D). Additionally, the negative zeta potential of Mal-LNP (−3.6 ± 0.6 mV) and Nb-LNP (−4.3 ± 0.4 mV) supports the suitability of these formulations for mRNA delivery applications (Figure 2E) [54]. Both Mal-LNP and Nb-LNP exhibited over 90% mRNA encapsulation efficiency (Figure 2F,H). To assess the stability of Mal-LNP and Nb-LNP, the formulations were kept at 4 °C and 25 °C for a duration of 7 days, during which no significant changes in particle size or PDI were observed (Figure 2G and Figure S2E).

**Figure 3:**
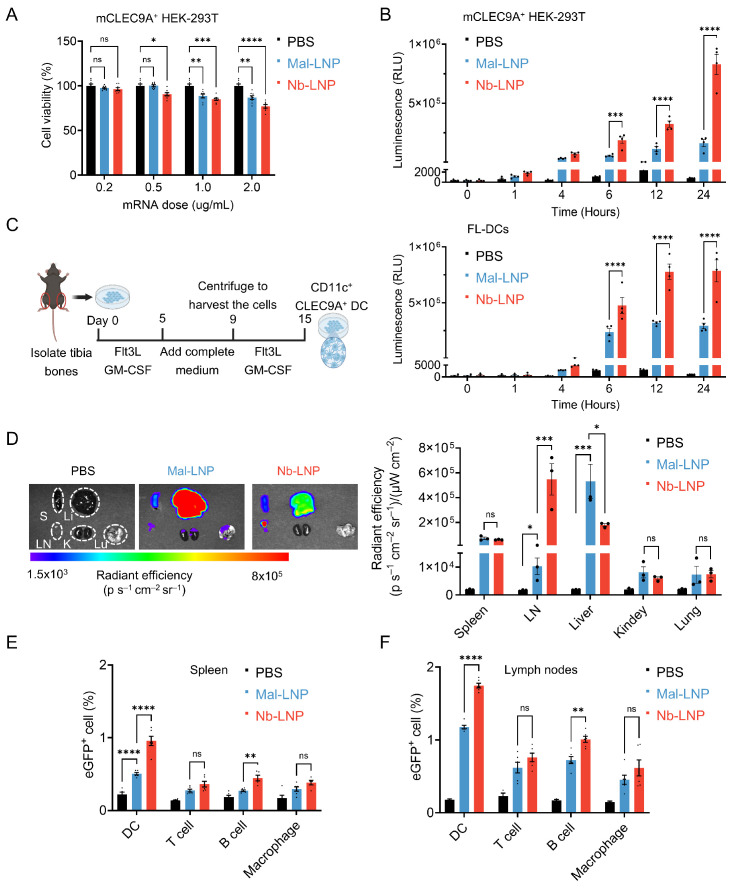
Transfection, cytotoxicity, and biodistribution of mRNA-LNP complexes. (**A**) Cytotoxicity of Mal-LNP and Nb-LNP in mCLEC9A^+^ HEK-293T cells. Cell viability evaluated using Cell Titer Luminescent Cell Viability Assay kit 24 h after treatment with mRNA Mal-LNP or Nb-LNP. (**B**,**C**) Time-course quantification of bioluminescence resulted from treating mCLEC9A^+^ HEK-293T cells and FL-DCs derived from mouse bone marrow with mRNA Mal-LNP or Nb-LNP at 0.5 μg/mL (*n* = 5). (**D**) Ex vivo imaging of organs was performed 4 h after injection. Shown are representative bioluminescence images and quantification of bioluminescence signals from organs extracted from C57BL/6 mice following intramuscular administration of 5 μg luciferase (Luc) formulated in either Mal-LNP or Nb-LNP. Abbreviations: LN, lymph node; Li, liver; S, spleen; Lu, lung; K, kidney. (**E**,**F**) Quantification of eGFP^+^ cells in distinct cell subsets in spleen and lymph nodes 36 h after mice were treated intramuscularly with 10 μg eGFP mRNA-LNP. Statistical analyses were performed using two-way ANOVA. All results are presented as mean ± SEM. “ns”, no significant difference; * *p* < 0.05; ** *p* < 0.01; *** *p* < 0.001; **** *p* < 0.0001.

The in vitro cytotoxicity of mRNA-loaded LNPs was assessed on mCLEC9A^+^ HEK-293T cells using an ATP cell viability luciferase assay. After 24 h incubation, both Mal-LNP and Nb-LNP showed only marginal cytotoxicity at up to 0.5 μg/mL (Figure 3A and Table S1). To assess whether Nb-LNP possesses a superior capacity to deliver mRNA to mCLEC9A-expressing cells, we treated mCLEC9A^+^ HEK-293T cells (Figure 3B and Figure S3A) and CLEC9A^+^ DCs derived from mouse bone marrow (Figure 3C and Figure S3B) with 0.5 μg/mL of luciferase mRNA-LNP. It was shown that luciferase expression became significantly higher 6 h post-transfection when mRNA was encapsulated by Nb-LNP compared to Mal-NLP in both mCLEC9A^+^ HEK-293T cells and CLEC9A^+^ DCs, with an approximately 3-fold difference after 24 h. To further evaluate the Nb-LNP’s efficacy of targeted delivery in vivo, C57BL/6 mice were subjected to intramuscular injection of 5 μg of luciferase mRNA-LNP, and bioluminescence signals were evaluated 4 h after injection. Notably, liver accumulation was significantly reduced with the use of Nb-LNP compared to Mal-LNP (Figure 3D). By contrast, bioluminescence intensity was markedly stronger with the use of Nb-LNP delivery (Figure 3D). Next, eGFP-encoding mRNA was used to evaluate the efficiency of mRNA-LNP uptake by immune cells in the spleen and lymph nodes. As expected, the population of eGFP^+^ DCs in both the spleen and lymph nodes was substantially higher by approximately 50% using Nb-LNP (Figure 3E,F and Figure S3C). Interestingly, a moderate increase in the eGFP^+^ B cell population was also observed with the use of Nb-LNP, although B cells are considered to be CLEC9A^-^ (Figure 3E,F).

### 3.3. Identification of Neoantigens in Mouse Lewis Lung Carcinoma

**Figure 4:**
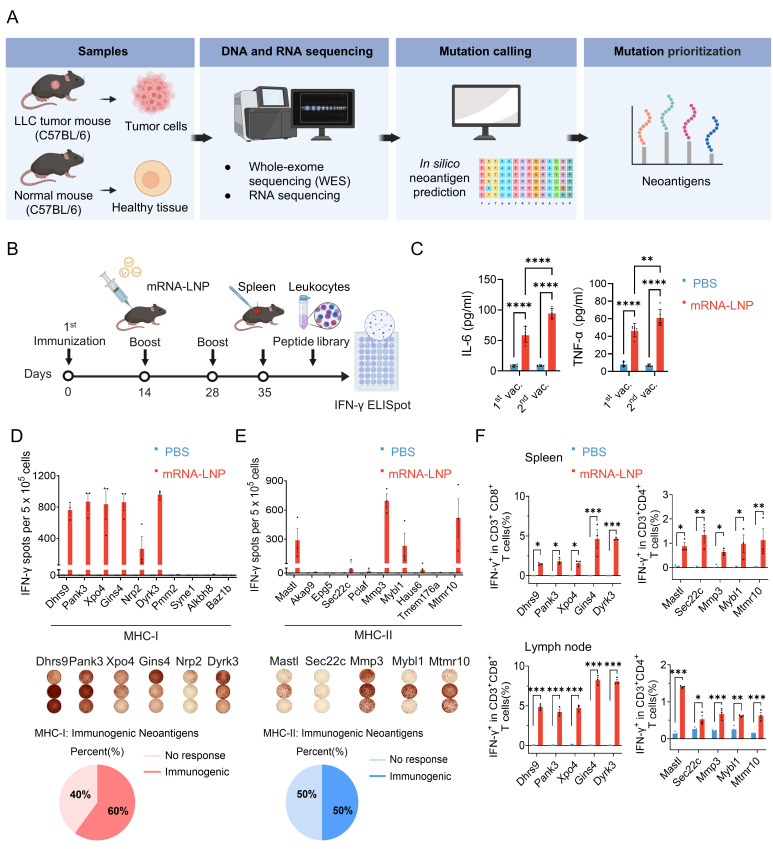
Construction of mRNA-LNP neoantigen vaccine and vaccine-induced T cell responses. (**A**) The workflow for identification of neoantigens in mouse Lewis lung carcinoma. (**B**) Schematic of immunization strategy in mice. (**C**) Serum levels of IL-6 and TNF-α were evaluated by using ELISA kits (*n* = 3). (**D**,**E**) Assessment of immunogenicity of mRNA-LNP vaccines in C57BL/6 mice (*n* = 3) by IFN-γ ELISpot assay. Mice were immunized with 10 μg of mRNA-LNP encoding each sequence of MHC class I (MHC-I) or class II (MHC-II) restricted neo-epitopes. The splenocytes were stimulated with each neo-epitope. (**F**) Secretion of each epitope-specific cytokine IFN-γ from CD8^+^ T cells and CD4^+^ T cells in spleen and lymph node cells. Data are presented as mean ± SEM. * *p* < 0.05; ** *p* < 0.01; *** *p* < 0.001; **** *p* < 0.0001.

Lung cancer remains the leading cause of cancer-related deaths worldwide, and the 5-year survival rate can be less than 20% in many countries [55]. Therefore, new therapies that can improve clinical outcomes in the treatment of lung cancer are urgently needed. In light of this, we chose mouse Lewis lung carcinoma (LLC) as the cancer model to assess whether Nb-LNP can confer mRNA cancer vaccines with superior therapeutic effects. Our first objective was to build the tumor mutanome and the neoantigen profile of LLC through next-generation sequencing (NGS) and machine learning algorithms (workflow outlined in Figure 4A). To this end, C57BL/6 wild-type background normal lung tissues and LLC cells were subjected to whole-exome sequencing (WES) and RNA sequencing (RNA-seq) to identify nonsynonymous single nucleotide substitutions (Methods and Figure S4). A total of 124 nonsynonymous mutations were identified (Table S2). Analysis with netMHCpan-4.0a and netMHCIIpan-4.0 showed that 42 and 10 of these were predicted to bind the MHC class I (H2-Db/Kb) and MHC class II (H2-IAb) alleles with a predicted affinity greater than 1000 nM, respectively (Tables S3 and S4).

### 3.4. Selection of Immunogenic Neo-Epitopes for Construction of mRNA Vaccine

Ten neo-epitopes were chosen based on their predicted affinity and encoded into two pentatope mRNAs (Tables S5 and S6). A low-affinity MHC class I-restricted peptide derived from Baz1b was randomly selected to assess whether predicted affinity correlated with induced T cell responses. C57BL/6 mice were immunized three times with 10 μg of mRNA-LNP (using Mal-LNP) encoding MHC class I- or MHC class II-restricted neoantigen epitopes (Figure 4B and Figure S5A,B), and peripheral blood was extracted for ELISA assay to detect the pro-inflammatory cytokines at 24 h post injection. The secretion of IL-6 and TNF-α was significantly increased after both the primary and booster immunization, which are critical mediators of anti-tumor T cell activation and recruitment (Figure 4C). The spleen cells of the immunized mice were used to examine the immunogenicity of the chosen neoantigens using an IFN-γ ELISpot assay. It was shown that the splenocytes were responsive to 6 out of the 10 MHC class I-restricted epitopes (Dhrs9, Pank3, Xpo4, Gins4, Nrp2 and Dyrk3) and 5 out of the 10 MHC class II-restricted epitopes (Mastl, Sec22c, Mmp3, Mybl1 and Mtmr10) (Figure 4D,E). These results were also observed in flow cytometric analysis. The five selected MHC class I-restricted epitopes (Dhrs9, Pank3, Xpo4, Gins4, and Dyrk3) and MHC class II-restricted epitopes (Mastl, Sec22c, Mmp3, Mybl1, and Mtmr10) could respectively induce the specific secretion of IFN-γ by CD8^+^ T cells or CD4^+^ T cells in both spleen and lymph nodes (Figure 4F), indicating that the identified neoantigens successfully stimulate the corresponding T cell pathways via MHC-I and MHC-II molecules, thereby confirming the accuracy and reliability of these predictive models.

### 3.5. Nb-LNP mRNA Vaccine Exhibits Superior Tumor Control and Favorable Safety

**Figure 5:**
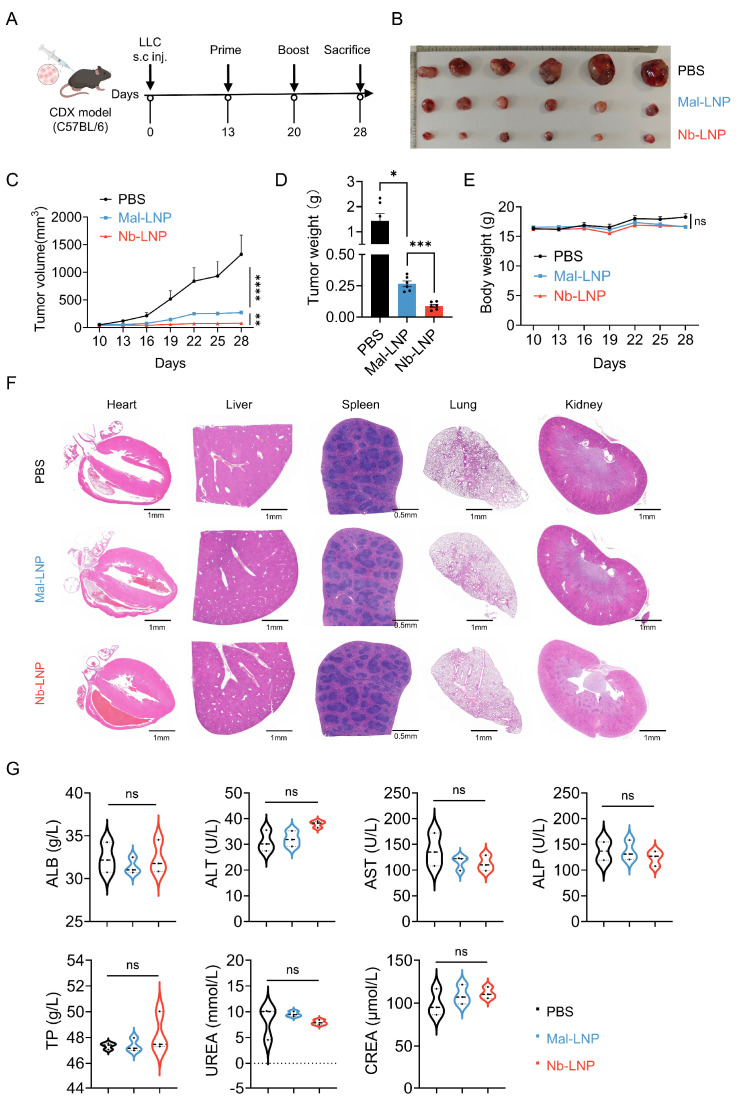
Therapeutic efficacy and in vivo safety profile of mRNA-LNP vaccines in a mouse LLC subcutaneous tumor model. (**A**) Schematic of building the mouse LLC subcutaneous tumor model and immunization strategy in mice (*n* = 6; 10 μg mRNA-LNP per mouse). (**B**) Surgically removed tumor tissues from mice 28 days after treatment (*n* = 6). (**C**) Measurement of tumor volume at different time-points after inoculation. (**D**) Measurement of tumor weight 28 days after treatment. (**E**) Measurement of body weight at different time-points after inoculation. (**F**) Hematoxylin and eosin (H&E) staining of major organs. Representative tissue sections of the heart, liver, spleen, lung, and kidney were examined 24 h following intramuscular administration of 10 μg of mRNA-LNP (*n* = 3). (**G**) Biochemical assessments of parameters indicative of organ function (albumin [ALB], alanine aminotransferase [ALT], aspartic acid aminotransferase [AST], alkaline phosphatase (ALP), total protein [TP], UREA, and creatinine (CREA) in mouse serum (*n* = 3)). Statistical analyses were performed using two-way ANOVA. Data are presented as mean ± SEM. “ns”, no significant difference, * *p* < 0.05, ** *p* < 0.01, *** *p* < 0.001, and **** *p* < 0.0001.

Five MHC class I- and MHC class II-restricted epitopes were chosen for the construction of the mRNA neoantigen vaccine used for in vivo anti-tumor studies. The mRNA encoding the MHC class I- and MHC class II-restricted epitopes were separately encapsulated in LNP and subsequently mixed in a 1:1 ratio. A cell line-derived xenograft (CDX) model was used, where C57BL/6 mice subcutaneously inoculated with LLC cells were subjected to a prime-boost regimen with 10 μg of mRNA-LNP encapsulated with Mal-LNP or Nb-LNP on day 13 and 20, respectively (Figure 5A and Figure S6A). Mice immunized with the Nb-LNP vaccine demonstrated significantly better tumor control compared to those treated with the Mal-LNP vaccine (Figure 5B–D), with an approximately 72% and 67% reduction in tumor volume and weight, respectively (Table S7). In contrast to the PBS control group where rapid tumor progression was observed, tumors in mice immunized with Mal-LNP or Nb-LNP became stable when a boost immunization was administered (Figure 5C and Figure S6B). In line with this, a slight increase in body weight was observed in control mice due to excessive tumor growth on day 28, although the difference was not significant (Figure 5E). To assess the in vivo safety profile of the Nb-LNP vaccine, hematoxylin and eosin (H&E) staining and a series of blood biochemical tests were performed. No distinguishable pathological change was observed in any of the major organs examined, including the heart, liver, spleen, lung and kidney (Figure 5F). In addition, no significant difference was found for any of the organ function parameters between the immunized and control groups (Figure 5G). These altogether suggest that there is no obvious adverse effect associated the mRNA neoantigen vaccines.

### 3.6. Nb-LNP mRNA Vaccine Induced Potent Cellular Immunity

**Figure 6:**
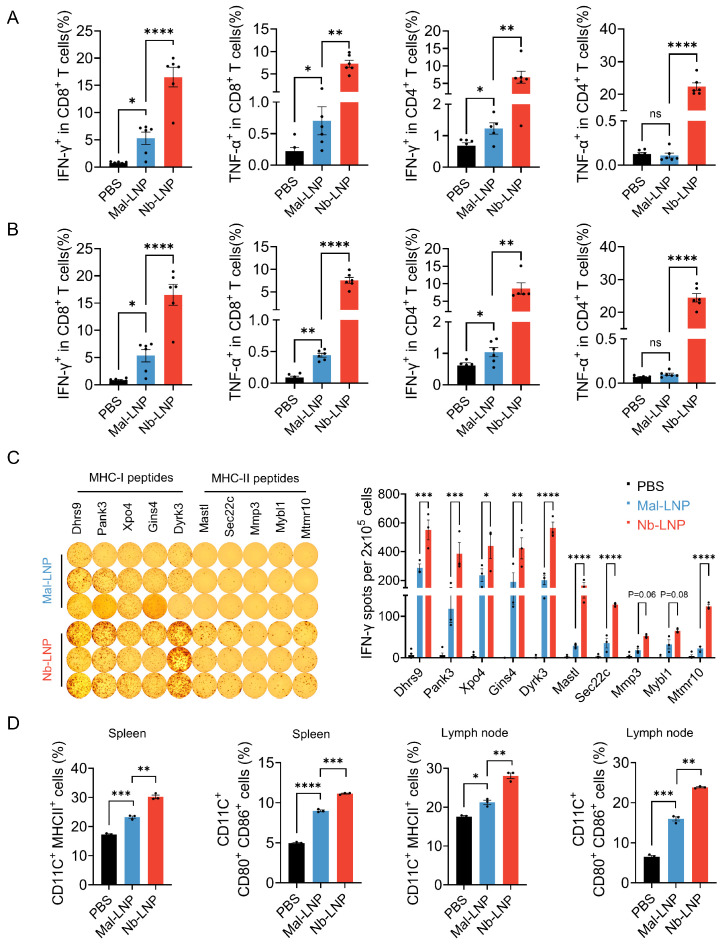
Enhanced cellular immune responses and DC maturation. (**A**) Quantification of neoantigen-specific IFN-γ^+^ or TNF-α^+^ CD8^+^ T cells and IFN-γ^+^ or TNF-α^+^ CD4^+^ T cells in spleen. (**B**) Quantification of neoantigen-specific IFN-γ^+^ or TNF-α^+^ CD8^+^ T cells and IFN-γ^+^ or TNF-α^+^ CD4^+^ T cells in lymph nodes. (**C**) Measurement of neoantigen-specific T cell responses by IFN-γ ELISpot assay. Left, ELISpot images; right, quantification of IFN-γ-producing T cells. Splenocytes were harvested on day 28 from mice immunized with a prime-boost regimen. (**D**) Assessment of DC maturation in the spleen and lymph nodes 36 h after intramuscular administration of a single dose of 10 μg mRNA Mal-LNP or Nb-LNP vaccine (*n* = 3). Statistical analyses were performed using two-way ANOVA. Data are presented as mean ± SEM. ns, no significant difference, * *p* < 0.05, ** *p* < 0.01, *** *p* < 0.001, and **** *p* < 0.0001.

To assess the T cell responses that drove the impressive anti-tumor effects, IFN-γ- and TNF-α-expressing CD4^+^ and CD8^+^ T cells in the spleen and lymph nodes of the immunized mice were examined. It was shown that all four T cell populations substantially expanded in response to treatment with Mal-LNP or Nb-LNP vaccines, with the latter being able to induce markedly stronger responses in all T cell subpopulations (Figure 6A,B and Figure S7). In both spleen and lymph nodes, an approximately 3-fold, 10-fold and 6-fold increase in IFN-γ^+^ CD8^+^ T cells, TNF-α^+^ CD8^+^ T cells and IFN-γ^+^ CD4^+^ T cells was observed respectively. Notably, while essentially no increase in TNF-α^+^ CD4^+^ T cells was observed in the Mal-LNP group, a roughly 170-fold and 350-fold expansion was found in the spleen and lymph nodes, respectively (Figure 6A,B and Table S8).

To assess whether all the selected neo-epitopes contributed to the induction of robust T cell responses, an ELISpot assay was conducted to quantify the amount of peptide-specific T cells (Figure 6C and Table S9). It was shown that all 10 peptide epitopes were able to trigger T cell responses, and the Nb-LNP vaccine elicited significantly higher levels than the Mal-LNP vaccine for all neo-epitopes except for MmP3 and Mybl1, which had stronger responses with the Mal-LNP, but the differences were not significant. Interestingly, the T cell responses triggered by MHC class II epitopes were lower than those elicited by MHC class I epitopes. Yet, the DC-targeted Nb-LNP vaccine was able to elicit roughly 2- to 6-fold augmented MHC class II-restricted T cell responses compared to the Mal-LNP vaccine. Tumor-infiltrating CD8^+^ and CD4^+^ T cells play a pivotal role in cancer immunotherapy. To investigate the tumor-infiltrating T cell populations, tumor-infiltrating lymphocytes (TILs) were isolated from vaccinated mice. Flow cytometric analysis revealed a significantly higher abundance of both CD8^+^ and CD4^+^ T cells in the Nb-LNP treatment group compared to the Mal-LNP group, exhibiting an approximate 2.2-fold increase (Figure S8A–C).

To confirm that the potent T cell responses observed were attributed to the targeted delivery of mRNA to DCs, thereby promoting the activation of DCs and presentation of epitopes to T cells, the spleen and lymph nodes of the vaccine-immunized mice were harvested to assess the expression of activation markers on the surface of DCs, including CD80, CD86 and MHC class II (Figure 6D and Figure S9) [56,57]. Indeed, the Nb-LNP vaccine was able to induce higher levels of DC maturation compared to the Mal-LNP vaccine, as evidenced by the significantly larger populations of CD11C^+^MHC class II^+^ cells and CD11C^+^CD80^+^CD86^+^ cells observed in both sites.

## 4. Discussion and Conclusions

While mRNA vaccines have shown great promise in combating infectious diseases and cancers, the development of more efficacious mRNA vaccines is confronted with various challenges, including targeted delivery to DCs. The mannose receptor (CD206, MR), an endocytic receptor found on DCs, facilitates the cross-presentation of soluble mannose ligands [29]. However, since the mannose receptor is also present on macrophages and endothelial cells, this may reduce the targeting specificity for DCs. Nanovaccines aimed at CLEC9A can directly activate T cells as artificial antigen-presenting cells and promote the maturation of DC subsets [58]. X-C Motif Chemokine Receptor 1 (XCR1), which binds the chemokine XCL1, is also selectively expressed on cDC1 [59]. By employing DEC-205 [60] and XCR1 as targeting molecules, it was observed that targeting XCR1 elicited a more pronounced IFN-γ^+^ CD8^+^ T cell response and increased cytotoxic activity within the spleen and lungs. Despite both CLEC9A and XCR1 being specific markers of the cDC1 subset, their engagement produced distinct immunological outcomes. Consequently, these findings suggest that the resultant effects and efficacy of targeting are influenced not only by the dendritic cell subtype but also by the specific receptor targeted.

In this study, we developed a precision-engineered Nb-LNP system where the LNP was incorporated with a nanobody binding with ultra-high affinity to CLEC9A, a receptor that is exclusively expressed on type 1 conventional dendritic cells (cDC1s), to promote DC-uptake of mRNA-LNP. Our results show that immunizing mice with luciferase mRNA encapsulated with Nb-LNP results in an augmented bioluminescence signal in lymph nodes (Figure 3D). Further, the Nb-LNP group demonstrates an approximately 50% larger population of eGFP^+^ DCs in both spleen and lymph nodes (Figure 3E,F). Unexpectedly, we also find that significantly higher proportion of eGFP^+^ B cells are present in both the spleen and lymph nodes. We reason that this may be due to the presence of large populations of B cells in the spleen and lymph nodes, and enrichment of mRNA Nb-LNP at these two sites may lead to increased non-specific uptake by B cells, which are also professional antigen-presenting cells capable of uptaking antigens.

Immunization of mRNA encoding MHC class I- and MHC class II-restricted neo-epitopes derived from mouse LLC using Nb-LNP as the delivery vehicle leads to higher levels of DC maturation compared to the use of Mal-LNP, as shown by substantially expanded populations of CD11C^+^MHC class II^+^ cells and CD11C^+^CD80^+^CD86^+^ cells (Figure 6D). After uptake of antigens, matured DCs migrate to lymph nodes to present antigens to T cells and initiate adaptive immune responses [61]. The intracellularly expressed epitopes encoded by mRNA can not only be presented on MHC class I molecules but also be presented on MHC class II molecules through autophagy and other non-autophagic pathways [62]. Indeed, we observe markedly more potent CD4^+^ and CD8^+^ T cell responses in the mRNA Nb-LNP group (Figure 6A,B). Notably, a large population of TNF-α^+^ CD4^+^ T cells emerges in response to mRNA Nb-LNP immunization, while essentially no expansion was observed in the mRNA Mal-LNP group (Figure 6A,B). Interestingly, IFN-γ^+^ T cell responses against MHC class II-restricted epitopes in Nb-LNP are remarkably higher than the Mal-LNP group by approximately 2- to 6-fold (Figure 6C). Driven by the more potent T cell responses, the mRNA neoantigen vaccine carried by Nb-LNP results in significantly better tumor control than Mal-LNP in the mouse LLC CDX model (Figure 5B–D).

The observation that immunization with Nb-LNP drives the phenomenally stronger CD4^+^ T cell responses is intriguing and has important implications. It is well known that CD4^+^ T cells play a vital role in adaptive immunity, as they not only form an important part of immune memory but also provide essential “help” to promote proliferation and differentiation of CD8^+^ T cells [63]. In addition, a subset of CD4^+^ T cells known as CD4^+^ cytotoxic lymphocytes (CTLs) can exert anti-tumor activity through direct cytotoxicity [64]. A precious study on T cell response in COVID-19 demonstrated association between dominance of SARS-CoV-2-specific TNF-α single-positive T cells and more durable anti-viral antibody responses, thus suggesting these cells may represent a pool of memory T cells [65]. Our observation of a substantially expanded TNF-α^+^ CD4^+^ T cell population in response to mRNA Nb-LNP immunization, which is approximately 4-fold larger than IFN-γ^+^ CD4^+^ T cells (Figure 6A,B), suggests that a similar TNF-α single-positive T cell population may also exist. This may lead to more durable memory immunity that accounts for more long-lasting tumor control. Further experiments are required to validate these speculations.

In effect, DCs are not only important in the induction of T cell responses but also in the elicitation of humoral responses. Priming with DCs is essential for differentiation of T follicular helper (Tfh) cells. Tfh cells play a crucial role in providing T cell help to B cells to facilitate the formation of germinal center, where B cells undergo somatic hypermutation and affinity maturation [66,67]. Therefore, DC-targeted LNPs also have significant implications for the development of anti-infectious disease mRNA vaccines where antibody responses are of particular importance [68].

Another interesting finding in this study is that targeting the LNP to DCs can promote enrichment of the LNP in lymph nodes while reducing liver accumulation (Figure 3D). Unmodified LNPs are typically found to accumulate in the liver and are internalized by hepatocytes, which not only reduces vaccine efficacy but also leads to toxicity, thus limiting the scope of clinical applications [69]. Therefore, modifications of LNP formulation aiming to reduce liver tropism are under intensive investigation [70,71,72]. By adjusting the lipid composition of LNPs, tissue-specific distribution can be achieved, offering advantages for scalable industrial production [73]. This physical targeting approach facilitates standardization; however, its targeting precision remains limited. In contrast, antibody-based modifications provide greater specificity but may introduce increased complexity and cost in the manufacturing process [74]. Although nanobodies present benefits over conventional antibodies, including smaller molecular weight and ease of modification [75], their conjugation to LNPs requires meticulous control. Given the complexity of the in vivo environment, comprehensive investigations are necessary to assess the stability of nanobody–LNP conjugates. Furthermore, optimizing the balance between conjugation efficiency and stability during large-scale production remains a critical challenge.

This study also presents certain limitations. The long-term immune memory elicited by mRNA vaccines as well as the potential risk of immune tolerance remain unassessed. The animal model employed is based on homologous mouse lung cancer, and its generalizability across diverse tumor mutation profiles has yet to be established. Future research should investigate multi-target combined delivery strategies, such as simultaneous targeting of CLEC9A and XCR1, to synergistically activate multiple immune pathways. Additionally, integrating ionizable lipid engineering could facilitate the development of a “dual-locked” LNP platform that confers both organ tropism and cell specificity, thereby enhancing delivery efficiency, lowering manufacturing complexity, and advancing clinical translation.

In summary, our study describes a precision-engineered Nb-LNP delivery system that is able to specifically target DCs. The impressive enhancement of T cell responses, in particular CD4^+^ T cell responses, and anti-tumor effects triggered by the mRNA neoantigen vaccine delivered by this Nb-LNP suggests this targeting delivery strategy has great potential in the development of mRNA vaccines, not only against cancers but also infectious diseases.

## Data Availability

All data generated or analyzed during this study are included in this manuscript. All relevant data are available from the authors.

## Supplementary Materials

The following supporting information can be downloaded at: https://www.mdpi.com/article/10.3390/vaccines14030239/s1, File S1: Uncropped immunoblot and SDS-PAGE images; Figure S1: Isolation and characterization of mCLEC9A-specific nanobodies. (A) Left, schematic of mCLEC9A extracellular domain (ECD) fused to the N terminus of hIgG1 Fc (mCLEC9A-Fc). Middle, SDS-PAGE/Coomassie blue staining of purified mCLEC9A-Fc which was used as the antigen for immunization of llama. Right, mCLEC9A-specific serum titers measured after the third immunization. (B) The CDR sequences of isolated mCLEC9A-specific nanobodies. (C) SDS-PAGE/Coomassie blue staining of purified anti-mCLEC9A nanobodies. (D) Measurement of binding affinity and kinetics of 2A3, 2C12, 2E11 and 2E12 to mCLEC9A by BLI. Two-fold serial dilutions of nanobodies from 100 nM to 6.25 nM were used to measure binding to biotinylated mCLEC9A immobilized on the Octet BLI biosensor. The equilibrium dissociation constant (*K*_D_) and binding kinetic parameters are presented; Figure S2: Conjugation of nanobody to Mal-LNP. (A) Schematic of the TCEP-reduced nanobody-HLC (a modified version of nanobody 2A4 added with a histidine, a hIgA1 hinge linker and cysteine at its C terminus) used for conjugation. (B) SEC elution profile of the purified nanobody-HLC and the SDS-PAGE/Coomassie blue staining of the two elution peaks under reducing and non-reducing conditions. (C) Schematic of conjugation of nanobody to Mal-LNP. (D) Native polyacrylamide gel electrophoresis followed by western blot detecting the nanobody to measure the amount of free nanobody in solution after functionalization. (E) In vitro stability of Nb-LNP and Mal-LNP at 25 °C for 7 days; Figure S3: Transfection and biodistribution of mRNA-LNP complexes. (A) Determination of mCLEC9A expression on transfected HEK-293T cells by flow cytometry. (B) Determination of mCLEC9A expression on FL-DCs by flow cytometry. (C) Quantification of eGFP^+^ cells in CD11c^+^CLEC9A^+^DCs in spleen and lymph nodes 36 h after mice were treated intramuscularly with 10 µg eGFP mRNA-LNP. Statistical analyses were performed using two-way ANOVA. All results are presented as mean ± SEM. * *p* < 0.05; ** *p* < 0.01; Figure S4: The workflow for identification of nonsynonymous single nucleotide substitutions and neoepitopes; Figure S5: Construction of mRNA-LNP neoantigen vaccine and vaccine-induced T cell responses. (A) Construction and sequence of MHC-I and MHC-II restricted neoantigens, each with five mutations (pentatope RNA). (B) Dose, administration route, and experimental group of neoantigen mRNA vaccines. (C) The assessment of mRNA quality and integrity was conducted using denaturing agarose gel; Figure S6: Measurement of tumor volume at different time-points after inoculation. (A) Dose, administration route, and experimental group of neoantigen mRNA vaccines. (B) Measurements of tumor volume for each individual mouse at different time-points after inoculation; Figure S7: Representative gating strategy for quantification of neoantigen-specific IFN-γ^+^ or TNF-α^+^ CD8^+^ T cells and IFN-γ^+^ or TNF-α^+^ CD4^+^ T cells by flow cytometry analysis; Figure S8: Representative flow cytometry plots (A) and quantitative percentages of infiltrating CD8^+^ T cells (B) and CD4^+^ T cells (C) in LLC tumors on day 28 (*n* = 6). * *p* < 0.05, ** *p* < 0.01, *** *p* < 0.001, and **** *p* < 0.0001; Figure S9: Representative gating strategy for evaluation of DC maturation in spleen and lymph nodes by flow cytometry analysis; Table S1: Evaluation of in vitro cytotoxicity by measurement of cell viability of mCLEC9A+ HEK-293T cells upon treatment with mRNA-LNPs; Table S2: List of nonsynonymous single nucleotide substitutions identified in mouse Lewis lung carcinoma; Table S3: List of MHC class I-restricted neo-epitopes; Table S4: List of MHC class II-restricted neo-epitopes; Table S5: MHC class I-restricted neo-epitopes selected for construction of mRNA neoantigen vaccine; Table S6: MHC class II-restricted neo-epitopes selected for construction of mRNA neoantigen vaccine; Table S7: Measurements of tumor volume and tumor weight of each individual mouse in different groups; Table S8: Average percentage of different subsets of neoepitope-specific T cells; Table S9: Quantification of neoantigen-specific T cell responses by ELISpot assay.
