# Supplementary material for: A Precision-Engineered DC-Targeting mRNA-LNP Neoantigen Vaccine Elicits Stronger T Cell Responses and Exhibits Superior Tumor Control
Source: Vaccines (Basel). 2026 Mar 5;14(3):239. doi: 10.3390/vaccines14030239 (PMC13030069; doi:10.3390/vaccines14030239)

Uncropped immunoblot images

Supplementary Figure S2D: Native polyacrylamide gel electrophoresis followed by western blot detecting the nanobody to measure the amount of free nanobody in solution after functionalization.

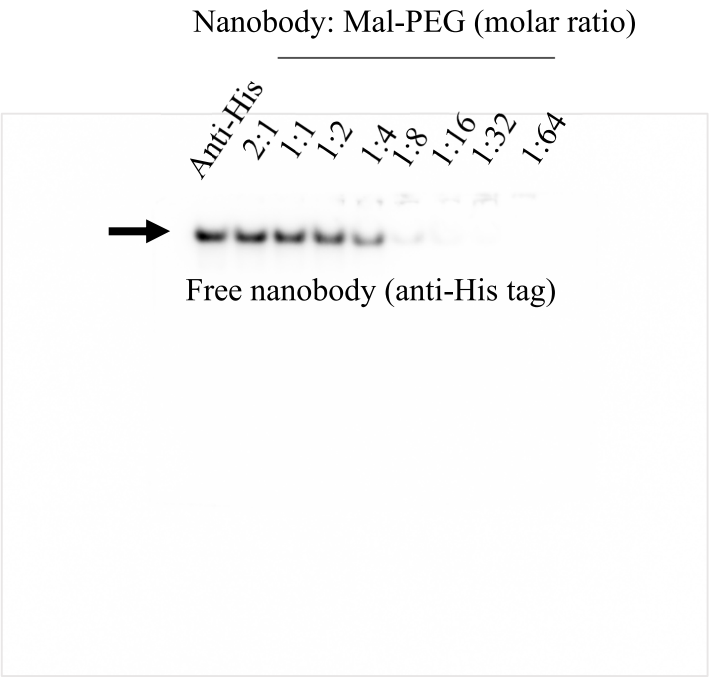

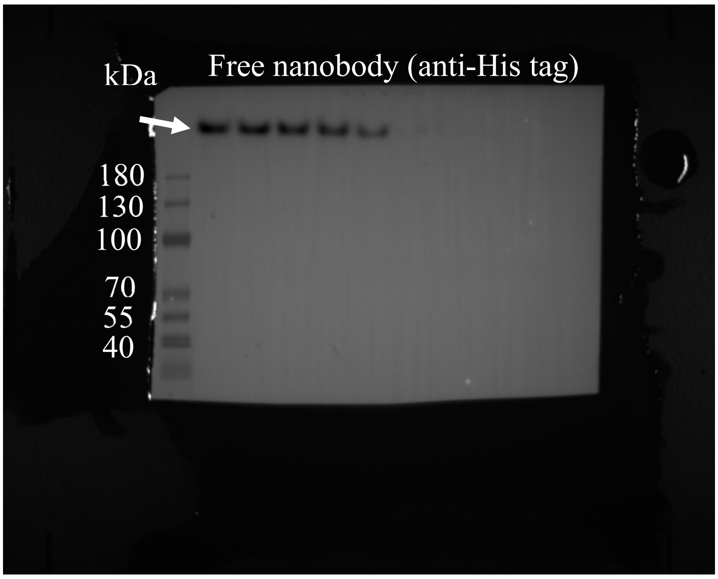

Uncropped Cyro-EM images

Figure 2D: Cryo-EM images of Mal-LNP and Nb-LNP. Scale bar indicated 50 nm.

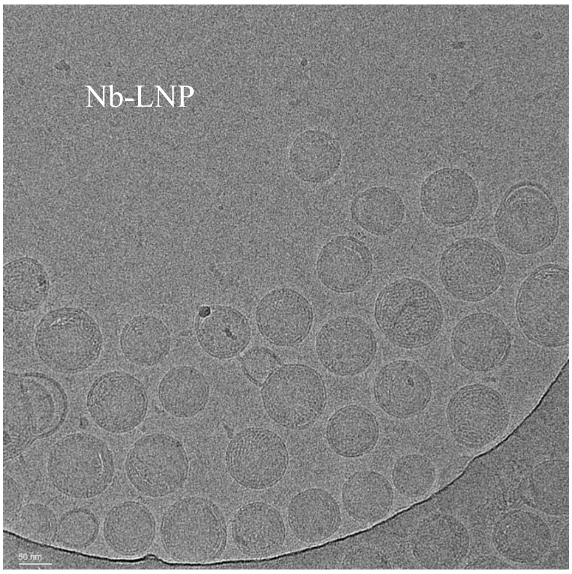

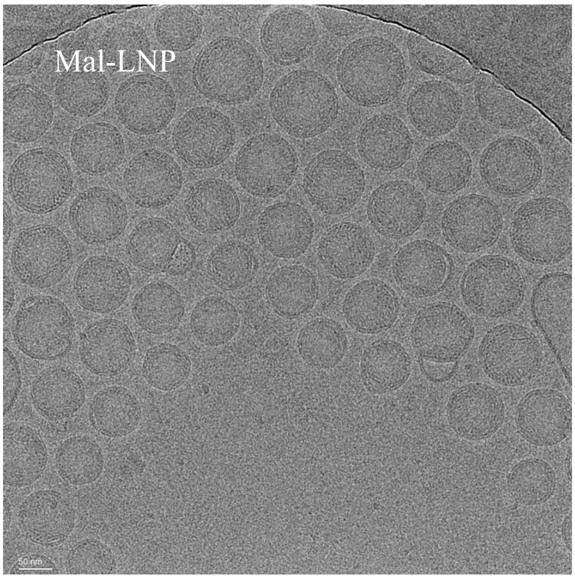

Uncropped SDS-PAGE images.

Figure 1B: SDS-PAGE/Coomassie blue staining of purified anti-mCLEC9A nanobody 2A4.

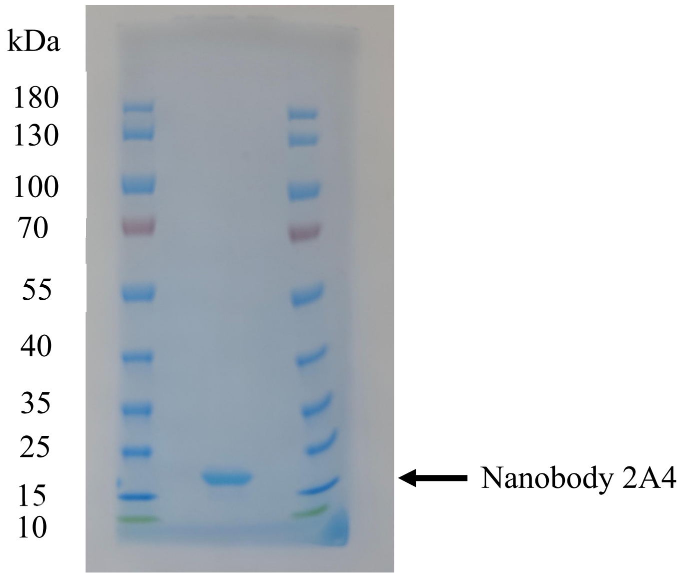

Figure 1C: SDS-PAGE/Coomassie blue staining of purified 2A4-Fc.

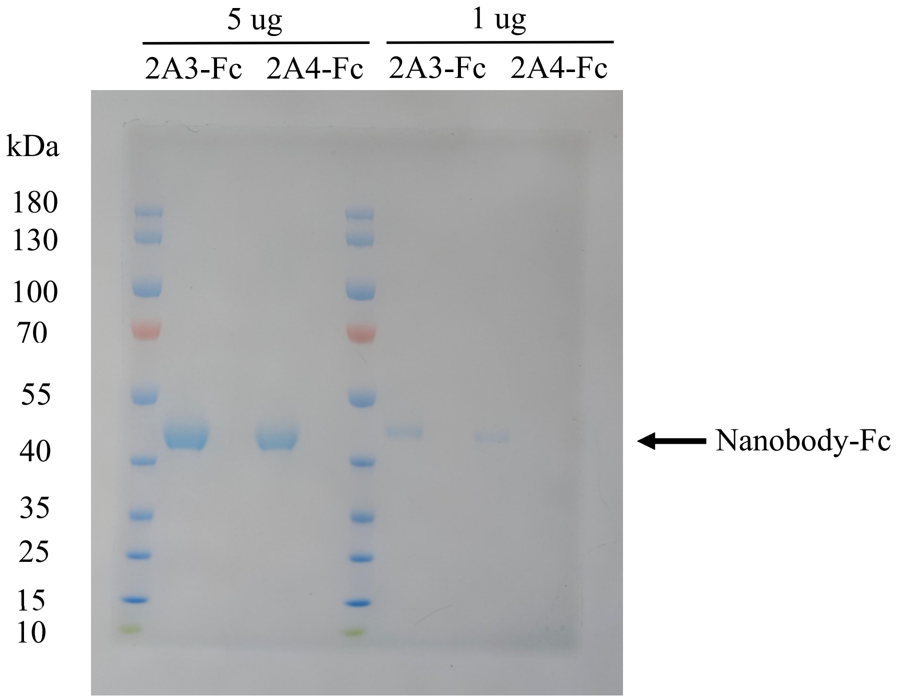

Figure S1A: SDS-PAGE/Coomassie blue staining of purified mCLEC9A-Fc which was used as the antigen for immunization of llama.

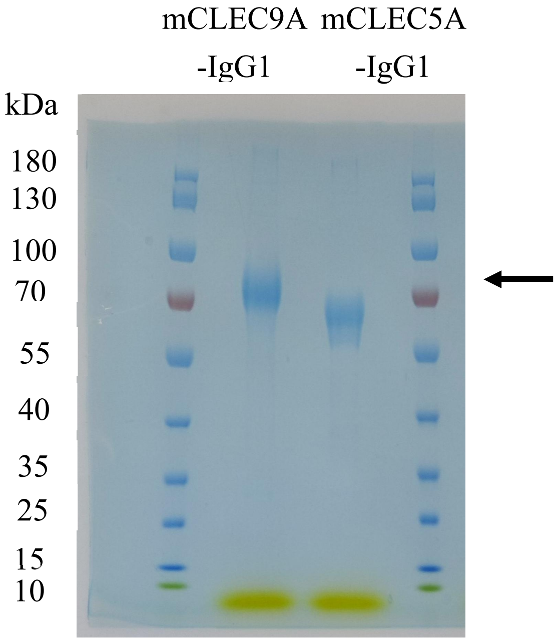

Figure S1C: SDS-PAGE/ Coomassie blue staining of purified anti-mCLEC9A nanobodies.

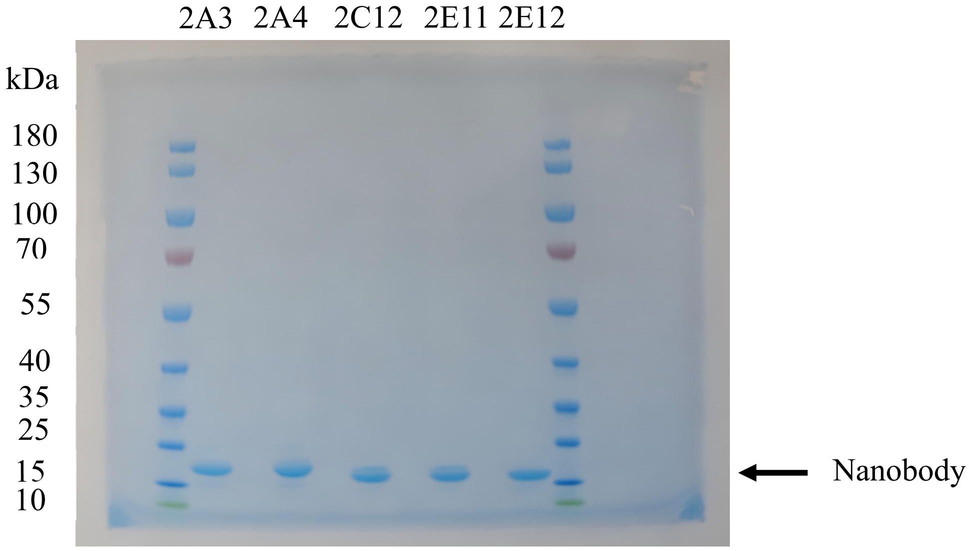

Figure S2B: SDS-PAGE/Coomassie blue staining of the two elution peaks under reducing and non-reducing conditions.

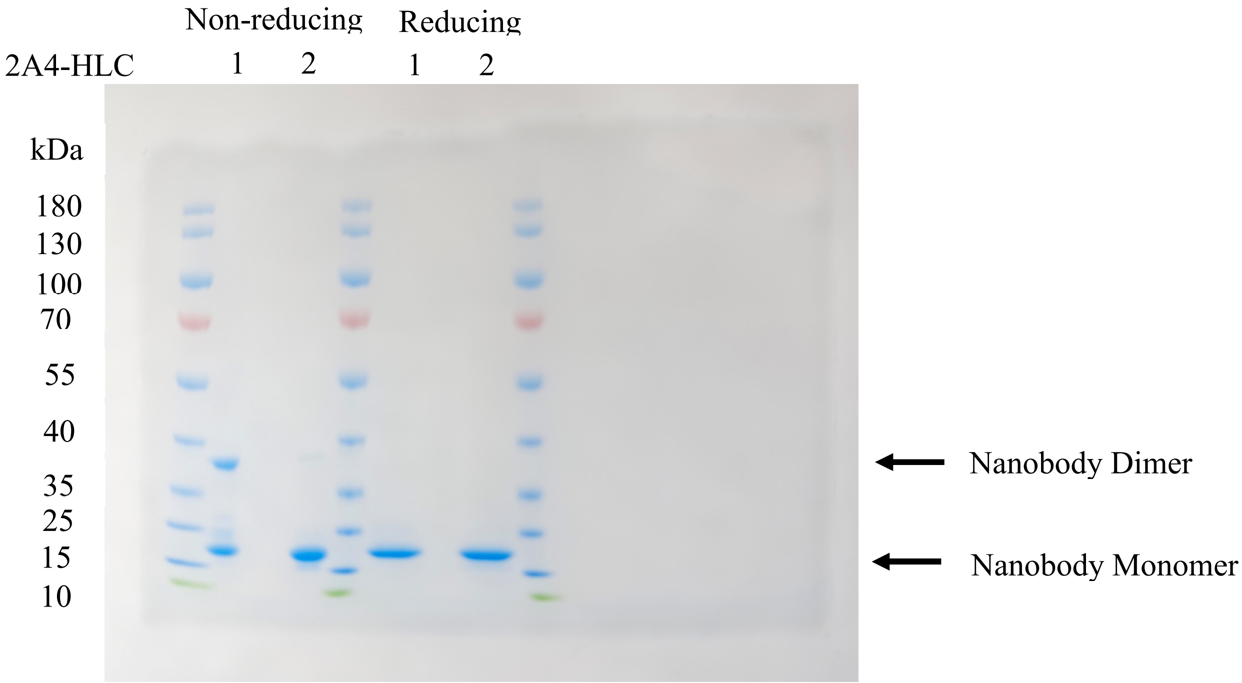

Supplement: Supplementary file 1 [file vaccines-14-00239-s001.zip › File S1. Uncropped immunoblot and SDS-PAGE images.pdf]
