# Supplementary material for: A Precision-Engineered DC-Targeting mRNA-LNP Neoantigen Vaccine Elicits Stronger T Cell Responses and Exhibits Superior Tumor Control
Source: Vaccines (Basel). 2026 Mar 5;14(3):239. doi: 10.3390/vaccines14030239 (PMC13030069; doi:10.3390/vaccines14030239)
Supplement: Supplementary file 1 [file vaccines-14-00239-s001.zip › vaccines-4139226-supplementary.pdf]

Supporting Information

A Precision-Engineered DC-Targeting mRNA-LNP Neoantigen Vaccine Elicits Stronger T Cell Responses and Exhibits Superior Tumor Control

Qi Liu [1,2], Yan Liu [1,2], Jinwei Li [1], Si Huang [1], Zhiying Chen [1], Jia Li [1,2], Tao Wang [3], Peipei Zhou [2], Jiandong Huo [1,2,*] and Dehua Li [1,*]

1 State Key Laboratory of Respiratory Disease, National Clinical Research Center for Respiratory Disease, Guangzhou Institute of Respiratory Health, The First Affiliated Hospital of Guangzhou Medical Uni-versity, Guangzhou 510120 , China; liu_qi@gzlab.ac.cn (Q.L.); liu_yan01@gzlab.ac.cn (Y.L.); 2023210030@stu.gzhmu.edu.cn (J.L.); 2023210031@stu.gzhmu.edu.cn (S.H.); 2023210460@stu.gzhmu.edu.cn (Z.C.); jiali@gzhmu.edu.cn (J.L.)

2 Guangzhou National Laboratory, Guangzhou International Bio Island, No. 9 XingDaoHuanBei Road, Guangzhou 510005, China; zhou_peipei@gzlab.ac.cn

3 GemPharmatech Co., Ltd., Foshan 528225, China; wangtao@gempharmatech.com

* Correspondence: huojiandong@gird.cn (J.H.); 2023390251@gzhmu.edu.cn (D.L.)

This file includes:

Figures S1–S9

Tables S1–S9

Figure S1

A

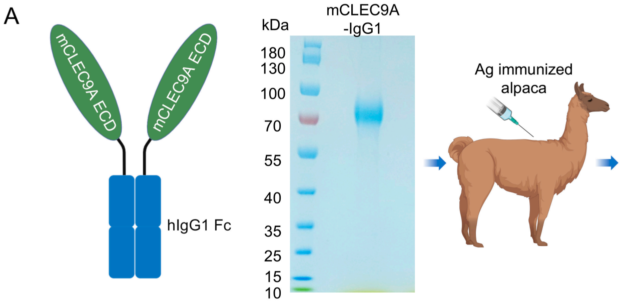

Dilution Factor	mCLEC9A 3rd immunization
2K	3.94
4K	3.96
8K	3.97
16K	3.95
32K	3.93
64K	3.76
128K	3.21
256K	2.80
BLANK	1.83

B

Nanobody ID	CDR1	CDR2	CDR3
2A3	GRDFSIYA	ITGGGTV	NAIDPINYKNLED
2A4	GTTFGIYA	ITRGGSE	NVVPPPFLKLDLTY
2C12	GSIFSDYA	ITRGGST	NAVDPFRYTPFGS
2E11	GSIFSTYA	ITSGGLA	NAVYPVPPYGPVGA
2E12	GSIFSIYA	ITAGGSA	NAIHPGEVTRPFDH

C

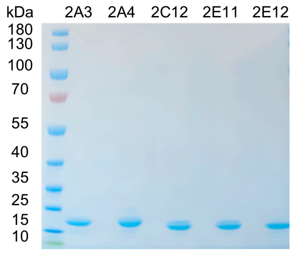

D

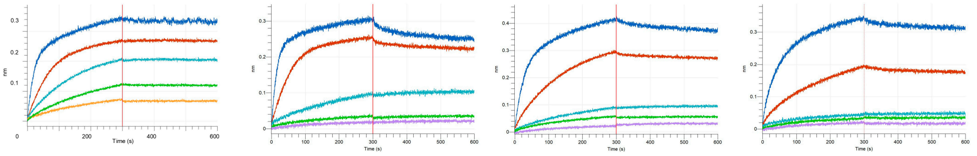

2A3 K_D(pM)	3.8
K_a(1/Ms)	1.3E+05
K_d (1/s)	4.9E-07

2C12 K_D(nM)	2.2
K_a(1/Ms)	1.9E+05
K_d (1/s)	4.2E-04

2E11 K_D(nM)	1.7
K_a(1/Ms)	9.7E+04
K_d (1/s)	1.7E-04

2E12 K_D(nM)	2.1
K_a(1/Ms)	8.7E+04
K_d (1/s)	1.8E-04

Figure S1. Isolation and characterization of mCLEC9A-specific nanobodies. (**A**) Left, schematic of mCLEC9A extracellular domain (ECD) fused to the N terminus of hIgG1 Fc (mCLEC9A-Fc). Middle, SDS-PAGE/Coomassie blue staining of purified mCLEC9A-Fc which was used as the antigen for immunization of llama. Right, mCLEC9A-specific serum titers measured after the third immunization. (**B**) The CDR sequences of isolated mCLEC9A-specific nanobodies. (**C**) SDS-PAGE/ Coomassie blue staining of purified anti-mCLEC9A nanobodies. (**D**) Measurement of binding affinity and kinetics of 2A3, 2C12, 2E11 and 2E12 to mCLEC9A by BLI. Two-fold serial dilutions of nanobodies from 100 nM to 6.25 nM were used to measure binding to biotinylated mCLEC9A immobilized on the Octet BLI biosensor. The equilibrium dissociation constant (K_D) and binding kinetic parameters are presented.

Figure S2

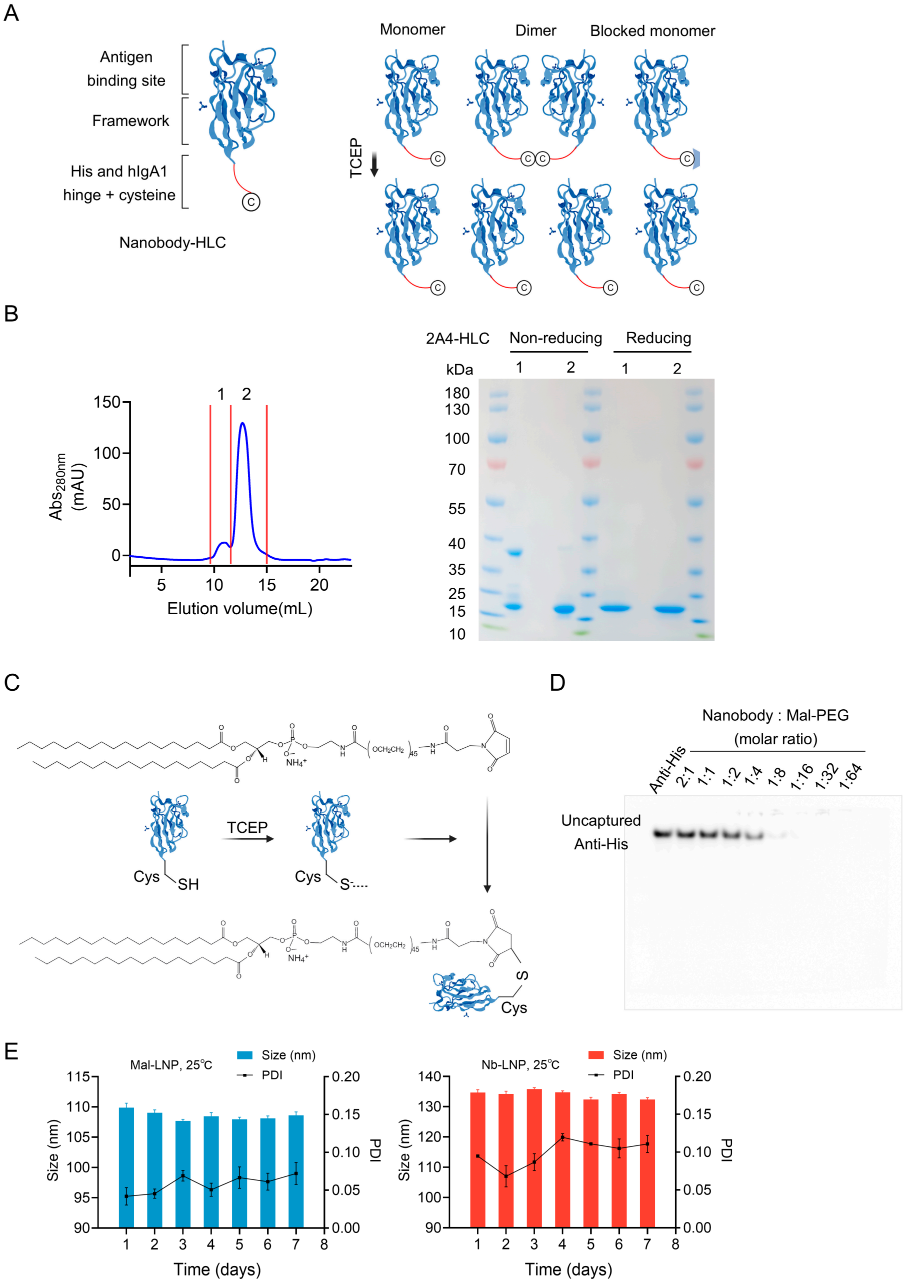

Figure S2. Conjugation of nanobody to Mal-LNP. (**A**) Schematic of the TCEP-reduced nanobody-HLC (a modified version of nanobody 2A4 added with a histidine, a hIgA1 hinge linker and cysteine at its C terminus) used for conjugation. (**B**) SEC elution profile of the purified nanobody-HLC and the SDS-PAGE/Coomassie blue staining of the two elution peaks under reducing and non-reducing conditions. (**C**) Schematic of conjugation of nanobody to Mal-LNP. (**D**) Native polyacrylamide gel electrophoresis followed by western blot detecting the nanobody to measure the amount of free nanobody in solution after functionalization. (**E**)

In vitro stability of Nb-LNP and Mal-LNP at 25°C for 7 days.

Figure S3

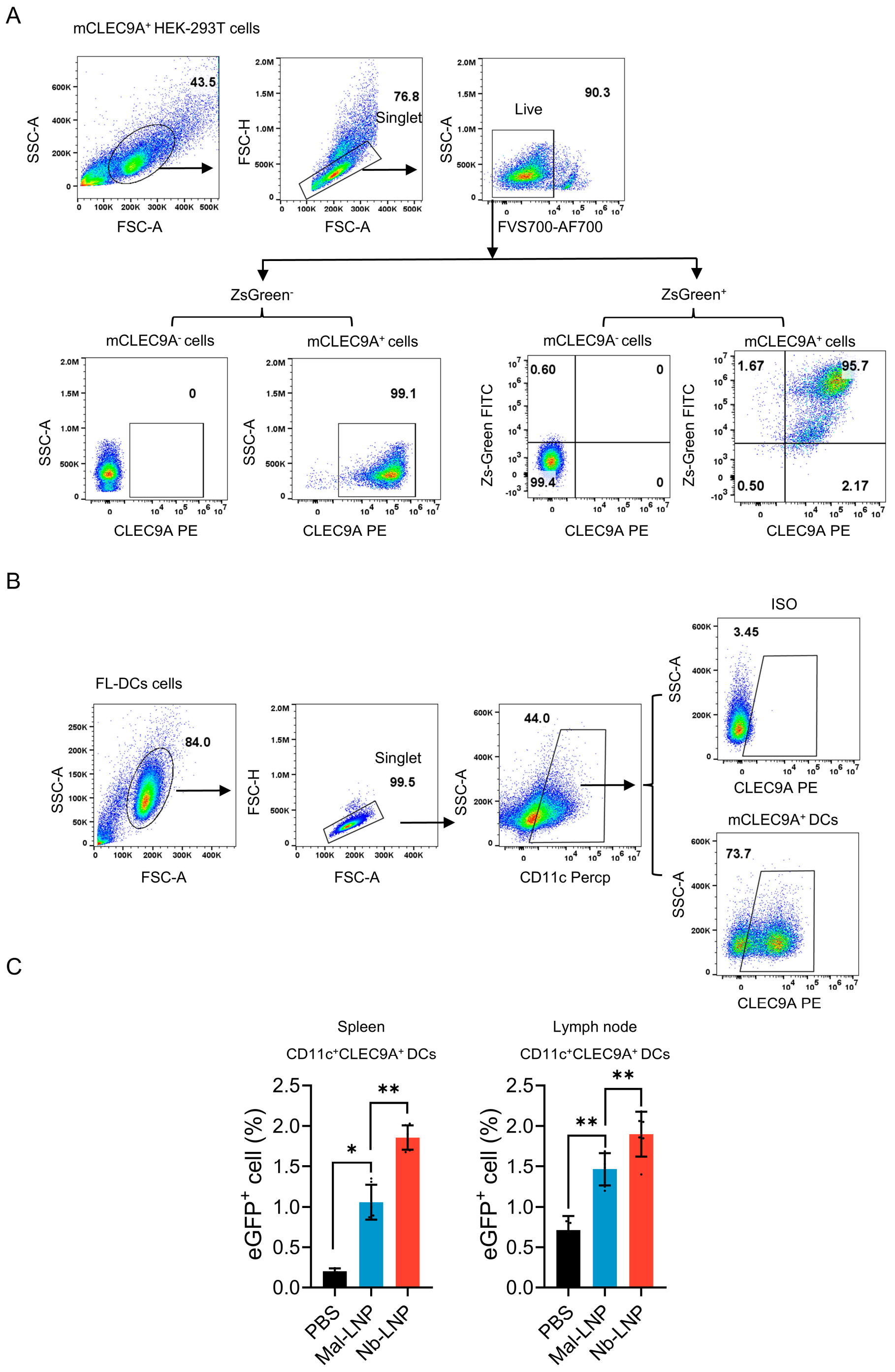

Figure S3. Transfection and biodistribution of mRNA-LNP complexes. (A) Determination of mCLEC9A expression on transfected HEK-293T cells by flow cytometry. (B) Determination of mCLEC9A expression on FL-DCs by flow cytometry. (C) Quantification of eGFP$^+$ cells in CD11c$^+$CLEC9A$^+$DCs in spleen and lymph nodes 36 hours after mice were treated intramuscularly with 10 μg eGFP mRNA-LNP. Statistical

analyses were performed using two-way ANOVA. All results are presented as mean ± SEM. *$p < 0.05$; **$p < 0.01$.

Figure S4

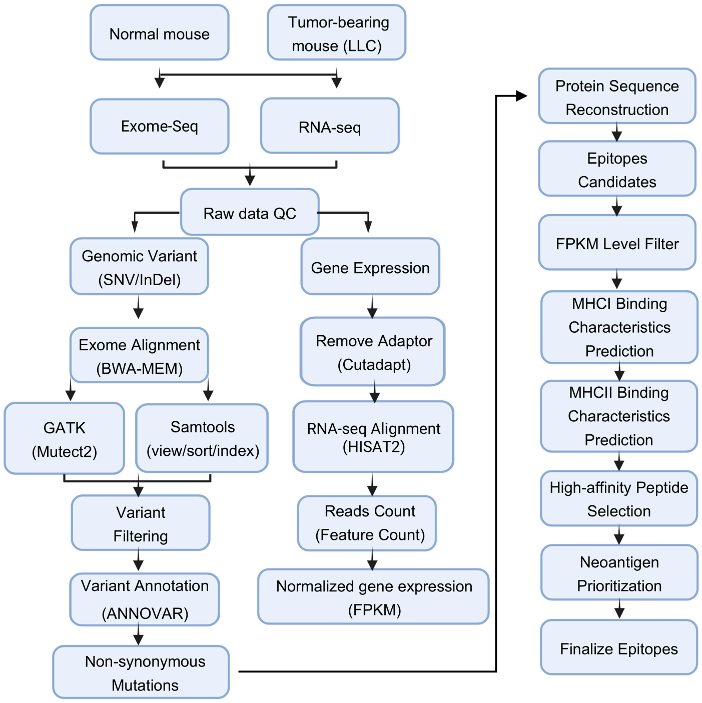

Figure S4. The workflow for identification of nonsynonymous single nucleotide substitutions and neoepitopes.

Figure S5

A

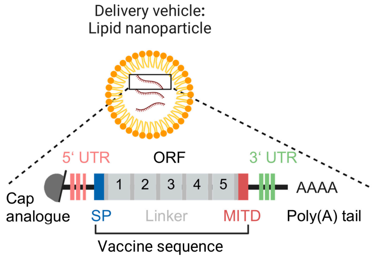

B

mRNA-LNP	Immune dose (ug)	Immunization route
PBS		Intramuscular
MHC-I	10	Intramuscular
MHC-II	10	Intramuscular

C

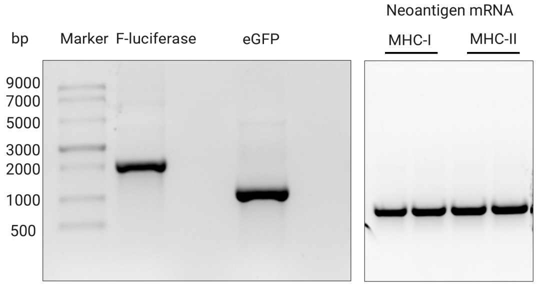

>MHC-I restricted neoantigens sequence

MDAMKRGLCCVLLLCGAVFVSPSPIEVNLFGLINVTPNMLPLVKKARGRVGGSGGGGSGGCAVNEKINRVVF

VWNFLRVNTLSMKLLGGSGGGGSGGYSRTDSVIRLLSALLRVSEVESRAIRAGGSGGGGSGGRRAKKGDLKV

SIHHMEMERIRYVLSSYGGSLGGGGSGNVIHMLESFTFRNNVCMAFELLSIDLYHHHHHHIVGIVAGLAVLA

VVVIGAVVATVMCRRKSSGGKGGSYSQAASSDSAQGSDVSLTA

>MHC-II restricted neoantigens sequence

MDAMKRGLCCVLLLCGAVFVSPSTKTELELALSPIHYSSAIPAAGSNQVTGGSGGGGSGGLEKIQEELEFQP

PEVLSLEDTDVANGMGGSGGGGSGGEPEFYLISSFWPSFPSNMDAAYEVTNRGGSGGGGSGGKNSRNGGDSE

AIPFTSPNVVKFSTPPTGGSLGGGGSGHSGAEQYFKEWFSIPANLHGIILPRLSHHHHHHIVGIVAGLAVLA

VVVIGAVVATVMCRRKSSGGKGGSYSQAASSDSAQGSDVSLTA

Figure S5. Construction of mRNA-LNP neoantigen vaccine and vaccine-induced T cell responses. (A) Construction and sequence of MHC-I and MHC-II restricted neoantigens, each with five mutations (pentatope RNA). (B) Dose, administration route, and experimental group of neoantigen mRNA vaccines. (C) The assessment of mRNA quality and integrity was conducted using denaturing agarose gel.

Figure S6

A

mRNA-LNP	Immune dose (ug)	Immunization route
PBS		Intramuscular
Mal-LNP	10	Intramuscular
Nb-LNP	10	Intramuscular

B

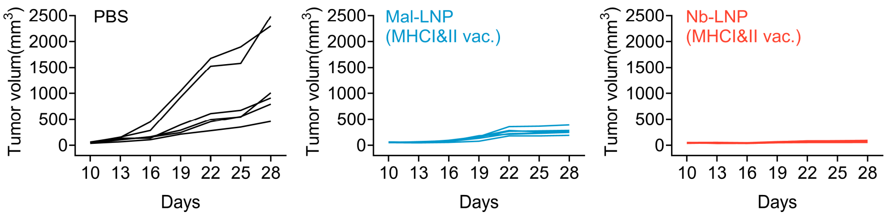

Figure S6. Measurement of tumor volume at different time-points after inoculation. (A) Dose, administration route, and experimental group of neoantigen mRNA vaccines. (B) Measurements of tumor volume for each individual mouse at different time-points after inoculation.

Figure S7

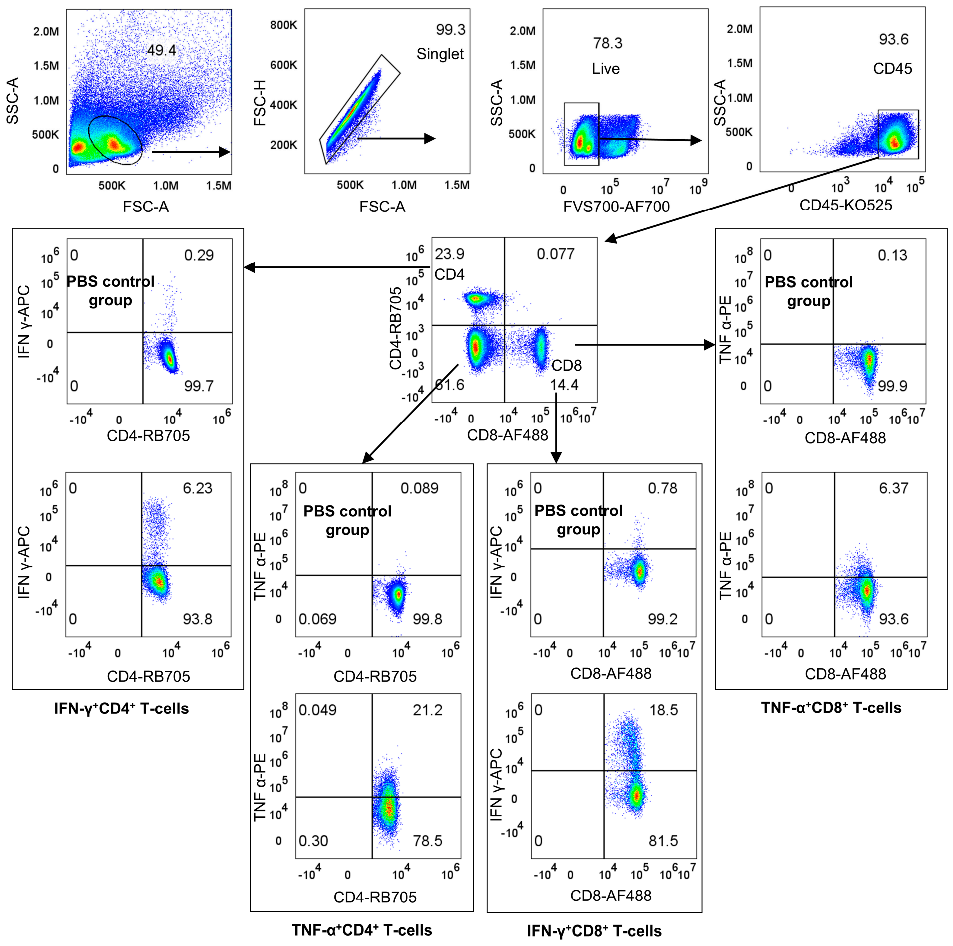

Figure S7. Representative gating strategy for quantification of neoantigen-specific IFN-γ^+ or TNF-α^+ CD8$^+$ T cells and IFN-γ^+ or TNF-α^+ CD4$^+$ T cells by flow cytometry analysis.

Figure S8

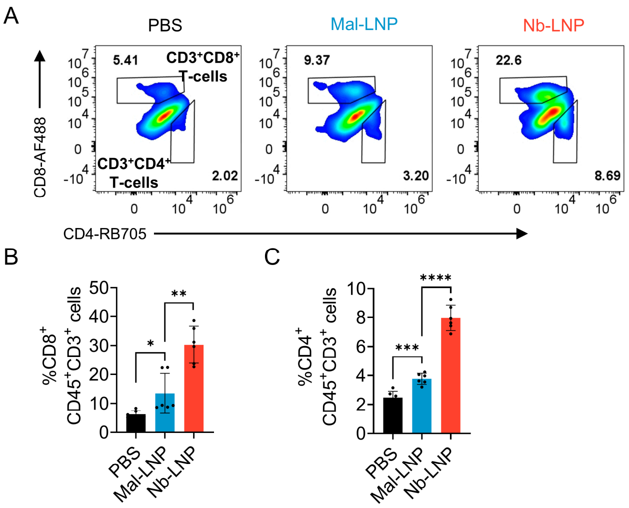

Figure S8. Representative flow cytometry plots (A) and quantitative percentages of infiltrating CD8⁺ T cells (B) and CD4⁺ T cells (C) in LLC tumors on day 28 (*n* = 6). * $p < 0.05$, ** $p < 0.01$, *** $p < 0.001$, and **** $p < 0.0001$.

Figure S9

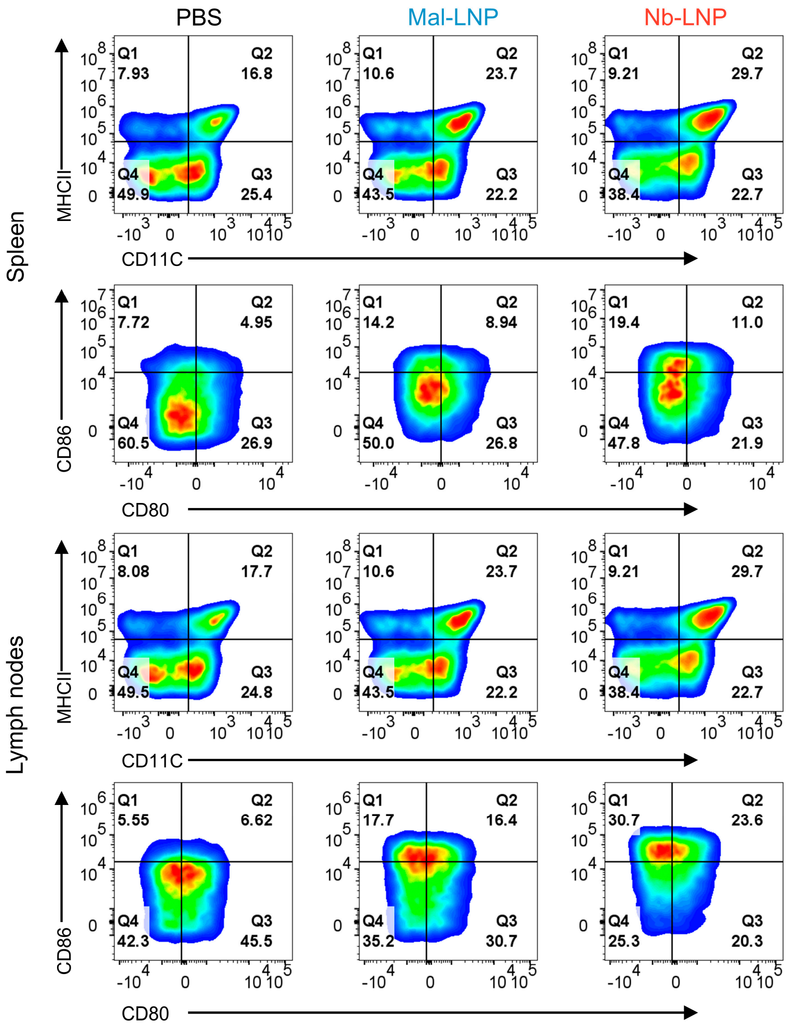

Figure S9. Representative gating strategy for evaluation of DC maturation in spleen and lymph nodes by flow cytometry analysis.

Table S1. Evaluation of *in vitro* cytotoxicity by measurement of cell viability of mCLEC9A+ HEK-293T cells upon treatment with mRNA-LNPs.

mRNA-LNP Concentration (ug/mL)	Cell viability (%mean)	
	Mal-LNP	Nb-LNP
0.2	97.60	96.36
0.5	100.13	90.58
1.0	88.66	84.93
2.0	86.85	77.09

Table S2. List of nonsynonymous single nucleotide substitutions identified in mouse Lewis lung carcinoma.

No	Refseq	MHC	Substitution (WT, AA#, Mut)	geneName
1	NM_001291857	H2-Kb	R976Q	Aebp1
2	NM_025630	H2-IAb	V610A	Aggf1
3	NM_194462	H2-Kb	D1478Y	Akap9
4	NM_001081264	H2-Kb	F231S	Alg6
5	NM_026303	H2-Kb	R625C	Alkbh8
6	NM_008569	H2-Kb	L773F	Anapc1
7	NM_001102430	H2-IAb	E145V	Arfgef1
8	NM_001085495	H2-Kb	A1737E	Arfgef2
9	NM_133674	H2-Kb	P465R	Arhgef5
10	NM_011714	H2-Kb	V523G	Baz1b
11	NM_007567	H2-IAb	P3350L	Bsn
12	NM_023223	H2-Kb	S408P	Cdc20
13	NM_026560	H2-Db	K80R	Cdca8
14	NM_001081363	H2-Kb	L292V	Cenpf
15	NM_007690	H2-IAb	E701G	Chd1
16	NM_181589	H2-Kb	F128S	Ckap2l
17	NM_181589	H2-Kb	I243V	Ckap2l
18	NM_181589	H2-IAb	T250A	Ckap2l
19	NM_175554	H2-IAb	L1285P	Clspn
20	NM_001110214	H2-Kb	F137L	Dclre1c
21	NM_175512	H2-Db	L146P	Dhrs9
22	NM_001159361	H2-Kb	V859A	Dip2b
23	NM_001277187	H2-IAb	I676V	Dlgap4
24	NM_001013371	H2-IAb	A561S	Dtx3l
25	NM_145508	H2-Db	H282N	Dyrk3
26	NM_001195633	H2-IAb	E1351G	Epg5
27	NM_001362612	H2-IAb	T103A	Ewsr1
28	NM_012056	H2-IAb	S386G	Fkbp9
29	NM_001167964	H2-Db	F150S	G2e3
30	NM_010295	H2-IAb	A626V	Gclc
31	NM_024240	H2-Kb	R79H	Gins4
32	NM_019827	H2-Kb	P136L	Gsk3b
33	NM_173400	H2-IAb	P850S	Haus6
34	NM_198298	H2-Kb	A836T	Helz
35	NM_008302	H2-IAb	K531N	Hsp90ab1

36	NM_001033228	H2-Kb	S182R	Itga1
37	NM_001033228	H2-Kb	R315S	Itga1
38	NM_008448	H2-Kb	A528P	Kif5b
39	NM_032396	H2-Kb	A424T	Kremen1
40	NM_001317353	H2-IAb	I310T	Lamp1
41	NM_001284523	H2-Kb	S177A	Larp4
42	NM_028782	H2-Kb	A405S	Lonp1
43	NM_173776	H2-Db	T149I	Maml2
44	NM_001029983	H2-Kb	P124L	Man1b1
45	NM_008549	H2-Kb	E996del	Man2a1
46	NM_008927	H2-Db	G202V	Map2k1
47	NM_025979	H2-Kb	D366Y	Mastl
48	NM_145955	H2-Kb	R189W	Mcmbp
49	NM_001193305	H2-IAb	D920H	Mical2
50	NM_010809	H2-Kb	L338F	Mmp3
51	NM_010809	H2-Kb	E307D	Mmp3
52	NM_001081323	H2-IAb	L307F	Mphosph9
53	NM_008625	H2-Kb	F920L	Mrc1
54	NM_021556	H2-Db	E254K	Mrps30
55	NM_010830	H2-Kb	K631E	Msh6
56	NM_172742	H2-Db	R633I	Mtmr10
57	NM_001290397	H2-Kb	L431F	Mybl1
58	NM_080837	H2-IAb	G65R	Mydgf
59	NM_022410	H2-IAb	W26C	Myh9
60	NM_146208	H2-Kb	M510V	Neil3
61	NM_146208	H2-Kb	C220R	Neil3
62	NM_146208	H2-Kb	V325A	Neil3
63	NM_023739	H2-IAb	N968H	Nfx1
64	NM_201232	H2-Kb	E796V	Nipbl
65	NM_029278	H2-Kb	V591M	Nop14
66	NM_008730	H2-Kb	E375Q	Nptx1
67	NM_011629	H2-Kb	T305I	Nr2c1
68	NM_010939	H2-Kb	W664L	Nrp2
69	NM_008740	H2-Db	I208V	Nsf
70	NM_146787	H2-Db	V9L	Olfr920
71	NM_133752	H2-Kb	G81A	Opa1
72	NM_145962	H2-Kb	G321W	Pank3
73	NM_183355	H2-IAb	S416A	Pbx1
74	NM_021567	H2-Db	K36N	Pcbp4
75	NM_026515	H2-Kb	R14S	Pclaf

76	NM_029404	H2-Kb	G261V	Phf14
77	NM_021280	H2-IAb	R243H	Plcg1
78	NM_177606	H2-Kb	N384T	Plekhh2
79	NM_177606	H2-Db	F191S	Plekhh2
80	NM_007408	H2-Kb	L183M	Plin2
81	NM_016881	H2-Kb	R119L	Pmm2
82	NM_026383	H2-Kb	H41L	Pnrc2
83	NM_009086	H2-Kb	M1069V	Polr1b
84	NM_016781	H2-Kb	R268L	Prkag1
85	NM_009012	H2-IAb	G200V	Rad50
86	NM_011240	H2-Kb	Y795H	Ranbp2
87	NM_025936	H2-Kb	D591N	Rars
88	NM_001204915	H2-Kb	T145R	Reep3
89	NM_178677	H2-IAb	A151E	Sec22c
90	NM_012032	H2-IAb	D470Y	Serinc3
91	NM_021506	H2-Kb	R452H	Sh3rf1
92	NM_016981	H2-Kb	P88L	Slc9a1
93	NM_001031814	H2-IAb	G3263D	Smg1
94	NM_146126	H2-Kb	L82F	Sord
95	NM_019442	H2-IAb	V168I	Stk19
96	NM_011505	H2-Kb	K109R	Stxbp4
97	NM_001347732	H2-Kb	F140V	Syne1
98	NM_001347732	H2-Kb	N7011I	Syne1
99	NM_001347732	H2-IAb	A6240P	Syne1
100	NM_001347732	H2-IAb	W605R	Syne1
101	NM_013763	H2-Db	S204N	Tbl2
102	NM_021297	H2-Kb	P33L	Tlr4
103	NM_001098271	H2-Kb	P96R	Tmem176a
104	NM_001098271	H2-IAb	V10I	Tmem176a
105	NM_175502	H2-Kb	L295F	Tmem74
106	NM_144925	H2-Kb	Q609L	Tnrc6a
107	NM_001083587	H2-Kb	S840A	Tns3
108	NM_176979	H2-Kb	K503E	Topbp1
109	NM_011640	H2-Kb	R334P	Trp53
110	NM_009448	H2-Kb	G10A	Tuba1c
111	NM_177185	H2-Kb	R551C	Ubn2
112	NM_025666	H2-IAb	P172A	Ubr7
113	NM_178648	H2-Kb	L179M	Ubxn8
114	NM_177151	H2-Kb	G3056R	Vps13b
115	NM_022997	H2-Kb	T390I	Vps35

116	NM_001355657	H2-Kb	V116M	Wdr3
117	NM_001355657	H2-IAb	K758R	Wdr3
118	NM_020506	H2-Kb	V611L	Xpo4
119	NM_172867	H2-Kb	M1334I	Zfp462
120	NM_001308323	H2-Kb	E21K	Zfp51
121	NM_183146	H2-Kb	A125S	Zfp729a
122	NM_178417	H2-Kb	E43D	Zfp867
123	NM_176962	H2-Kb	G172R	Zfp944
124	NM_001177568	H2-IAb	K150T	Zfp970

WT, wild type; AA#, position of mutated amino acid; Mut, mutation.

Table S3. List of MHC class I-restricted neo-epitopes.

No	Refseq	Peptide	27_mer sequence	nM	MHC	Substitution (WT, AA#, Mut)	geneName
1	NM_146787	MALINGSVL		6.89	H2-Db	V9L	Olfr920
2	NM_175512	FGLINVTPNM	PIEVNLFGLINVTPNMLPLVKKARGRV	8.15	H2-Db	L146P	Dhrs9
3	NM_145962	VVFVWNFL	CAVNEKINRVVFVWNFLRVNTLSMKLL	15.87	H2-Kb	G321W	Pank3
4	NM_020506	SVIRLLSAL	YSRTDSVIRLLSALLRVSEVESRAIRA	27.46	H2-Kb	V611L	Xpo4
5	NM_024240	VSIHHMEM	RRAKKGDLKVSIHHMEMERIRYVLSSY	54.47	H2-Kb	R79H	Gins4
6	NM_010939	WVYDHAKL	PEETCGWVYDHAKLLRSTWISSANPND	54.77	H2-Kb	W664L	Nrp2
7	NM_145508	FTFRNNVCM	NVIHMLESFTFRNNVCMAFELLSIDLY	73.02	H2-Db	H282N	Dyrk3
8	NM_016881	TFIEFLNGM	IKLPKKRGTFIEFLNGMLNVSPIGRSC	83.52	H2-Kb	R119L	Pmm2
9	NM_001347732	TIILYVQI	SIVLGLMWTIILYVQIEELTSNLPQLQ	97.61	H2-Kb	F140V	Syne1
10	NM_026303	VFHCYYHV	GVAGCPDPSPVFHCYYHVFCDGELEAS	100.45	H2-Kb	R625C	Alkbh8
11	NM_172867	YTYIATKL	QRRHPEHYVDYTYIATKLWAGPDPSSP	110.83	H2-Kb	M1334I	Zfp462
12	NM_026560	FALGGNRQAL	GMKWLDYFALGGNRQALEEAAKADRDI	137.88	H2-Db	K80R	Cdca8
13	NM_021297	LNITYQCM	LTPGSLNPCIEVVLNITYQCMDQKLSK	141.45	H2-Kb	P33L	Tlr4
14	NM_016781	LSHYFEGV	NNLDVSVTKALQHLSHYFEGVLKCYLH	147.42	H2-Kb	R268L	Prkag1
15	NM_172742	FSIPANLHGI	HSGAEQYFKEWFSIPANLHGIILPRLS	153.85	H2-Db	R633I	Mtmr10
16	NM_001098271	AMYSEGARF	YIGHYLAMYSEGARFWTGIVAMLAGAV	176.15	H2-Kb	P96R	Tmem176a
17	NM_133752	LSPIKYAYQ	LSLHKLKLSPIKYAYQPRRNFWPARLA	188.09	H2-Kb	G81A	Opa1
18	NM_178417	VMEDVFRNL	DPSQKNLYRDVMEDVFRNLTSIEKGWE	203.26	H2-Kb	E43D	Zfp867
19	NM_025979	LSPIHYSSAI	TKTELELALSPIHYSSAIPAAGSNQVT	306.65	H2-Kb	D366Y	Mastl
20	NM_008549	KKSPVSYPSL	ILLEKRSAVNMEEKKSPVSYPSLLSHM	314.31	H2-Kb	E996del	Man2a1

21	NM_008730	ATQAFVGQL	LGGGFDATQAFVGQLAHFNIWDRKLTP	353.14	H2-Kb	E375Q	Nptx1
22	NM_021567	SIIGKNGETV	LMHGKEVGSIIGKNGETVKRIREQSSA	353.46	H2-Db	K36N	Pcbp4
23	NM_001159361	VFYDERIVAV	VFSVSVFYDERIVAVAEQRPDASEEDS	367.31	H2-Kb	V859A	Dip2b
24	NM_177151	GAFPRHQKL	AFVDAEIRLGAFPRHQKLCQFCISSMV	379.29	H2-Kb	G3056R	Vps13b
25	NM_183146	CSRSFHSPSL	LYIGMNPNKCEECSRSFHSPSLSSEEN	385.27	H2-Kb	A125S	Zfp729a
26	NM_001110214	VLYTGDLRL	FQGSNGTVLYTGDLRLAKGEASRMELL	389.54	H2-Kb	F137L	Dclre1c
27	NM_008569	LVYEEFKL	PAIFFVLHLVYEEFKLNTLMGEGICSL	392.81	H2-Kb	L773F	Anapc1
28	NM_008448	KSPTLASI	EYELLSDELNQKSPTLASIDAELQKLK	412.73	H2-Kb	A528P	Kif5b
29	NM_178648	ISPAARPLM	KFSTEISPAARPLMRKEVPDLPEEPSE	419.72	H2-Kb	L179M	Ubxn8
30	NM_010809	SFWPSFPSNM	EPEFYLISSFWPSFPSNMDAAYEVTNR	458.95	H2-Kb	L338F	Mmp3
31	NM_016981	LSRKAFPVL	HNLTNLIIEHGGKLSRKAFPVLDIDYP	468.97	H2-Kb	P88L	Slc9a1
32	NM_001033228	RIYPWESV	TQLDIVIVLDGSNRIYPWESVTAFLND	469.13	H2-Kb	S182R	Itga1
33	NM_022997	LNLEHIAI	VEIFNKLNLEHIAISSAVSKELTRLLK	471.90	H2-Kb	T390I	Vps35
34	NM_146126	KVGEFVKHL	GHEAAGTVTKVGEFVKHLKPGDRVAIE	497.42	H2-Kb	L82F	Sord
35	NM_007408	MLVDQYFPL	MNSGVDNAITKSEMLVDQYFPLTQEEL	543.80	H2-Kb	L183M	Plin2
36	NM_001081363	KMTELEVRL	QNQDLKSKMTELEVRLQGQEKEMRSQV	660.14	H2-Kb	L292V	Cenpf
37	NM_013763	IMTASNDTTV	GIADTGKFIMTASNDTTVLIWNLKGQV	667.69	H2-Db	S204N	Tbl2
38	NM_011240	KSYKHSPKT	PTKYSLSPSKSYKHSPKTPPRWAEDQN	731.74	H2-Kb	Y795H	Ranbp2
39	NM_011714	LGQKRYELL	EDRARLPEELRALGQKRYELLEHKKRW	750.69	H2-Kb	V523G	Baz1b
40	NM_173776	IQQRNTTQL	PGTYNVTSTMNQLIQQRNTTQLITNQN	753.02	H2-Db	T149I	Maml2
41	NM_146208	VHHRRCVL	GHHSKSDGSPLCKVHHRRCVLRVVRKD	815.96	H2-Kb	M510V	Neil3
42	NM_021556	YSIPTKIPV	LPEFVPLDYSIPTKIPVMKCKPDKLPL	979.83	H2-Db	E254K	Mrps30

Table S4. List of MHC class II-restricted neo-epitopes.

No	Refseq	Peptide	27_mer sequence	nM	MHC	Substitution (WT, AA#, Mut)	geneName
1	NM_025979	LSPIHYSSAIPAAG	TKTELELALSPIHYSSAIPAAGSNQVT	67.80	H2-IAb	D366Y	Mastl
2	NM_194462	GEYRKAVPLSSHDY	GEYRKAVPLSSHDYLDDILKSEEHGLA	174.44	H2-IAb	D1478Y	Akap9
3	NM_001195633	GVADFHYAASKALR	HVNLLKDMKRRLTGVADFHYAASKALR	287.02	H2-IAb	E1351G	Epg5
4	NM_178677	EELEFQPPEVLSLE	LEKIQEELEFQPPEVLSLEDTDVANGM	295.13	H2-IAb	A151E	Sec22c
5	NM_026515	GAYSKAVASQAPRK	MVRTKANYVPGAYSKAVASQAPRKVLG	299.78	H2-IAb	R14S	Pclaf
6	NM_010809	ISSFWPSFPSNMDA	EPEFYLISSFWPSFPSNMDAAYEVTNR	429.22	H2-IAb	L338F	Mmp3
7	NM_001290397	AIPFTSPNVVKFST	KNSRNGGDSEAIPFTSPNVVKFSTPPT	515.88	H2-IAb	L431F	Mybl1
8	NM_173400	HKVELSSVAKAVQA	NPKTLEQHKVELSSVAKAVQADDAHTV	735.49	H2-IAb	P850S	Haus6
9	NM_001098271	GARFWTGIVAMLAG	YIGHYLAMYSEGARFWTGIVAMLAGAV	829.18	H2-IAb	P96R	Tmem176a
10	NM_172742	YFKEWFSIPANLHG	HSGAEQYFKEWFSIPANLHGIILPRLS	889.43	H2-IAb	R633I	Mtmr10

Table S5. MHC class I-restricted neo-epitopes selected for construction of mRNA neoantigen vaccine.

Pentatope	Peptide	27_mer sequence	nM	Substitution (WT, AA#, Mut)	Gene Name
1	FGLINVT**P**NM	PIEVNLFGLINVT**P**NMLPLVKKARGRV	8.15	L146P	Dhrs9
1	VVFV**W**NFL	CAVNEKINRVVFV**W**NFLRVNTLSMKLL	15.87	G321W	Pank3
1	SVIRLLSA**L**	YSRTDSVIRLLSA**L**LRVSEVESRAIRA	27.46	V611L	Xpo4
1	VSIH**H**MEM	RRAKKGDLKVSIH**H**MEMERIRYVLSSY	54.47	R79H	Gins4
1	WVYDHAK**L**	PEETCGWVYDHAK**L**LRSTWISSANPND	54.77	W664L	Nrp2
2	FTFRN**N**VCM	NVIHMLESFTFRN**N**VCMAFELLSIDLY	73.02	H282N	Dyrk3
2	TFIEF**L**NGM	IKLPKKRGTFIEF**L**NGMLNVSPIGRSC	83.52	R119L	Pmm2
2	TIILY**V**QI	SIVLGLMWTIILY**V**QIEELTSNLPQLQ	97.61	F140V	Syne1
2	VFH**C**YYHV	GVAGCPDPSPVFH**C**YYHVFCDGELEAS	100.45	R625C	Alkbh8
2	L**G**QKRYELL	EDRARLPEELRAL**G**QKRYELLEHKKRW	750.69	V523G	Baz1b

Table S6. MHC class II-restricted neo-epitopes selected for construction of mRNA neoantigen vaccine.

Pentatope	Peptide	27_mer sequence	nM	Substitution (WT, AA#, Mut)	Gene Name
3	LSPIH**Y**SSAIPAAG	TKTELELALSPIH**Y**SSAIPAAGSNQVT	67.80	D366Y	Mastl
3	GEYRKAVPLSSHD**Y**	GEYRKAVPLSSHD**Y**LDDILKSEEHGLA	174.44	D1478Y	Akap9
3	**G**VADFHYAASKALR	HVNLLKDMKRRLT**G**VADFHYAASKALR	287.02	E1351G	Epg5
3	EELEFQPP**E**VLSLE	LEKIQEELEFQPP**E**VLSLEDTDVANGM	295.13	A151E	Sec22c
3	GAY**S**KAVASQAPRK	MVRTKANYVPGAY**S**KAVASQAPRKVLG	299.78	R14S	Pclaf
4	ISSFWPS**F**PSNMDA	EPEFYLISSFWPS**F**PSNMDAAYEVTNR	429.22	L338F	Mmp3
4	AIP**F**TSPNVVKFST	KNSRNGGDSEAIP**F**TSPNVVKFSTPPT	515.88	L431F	Mybl1
4	HKVELS**S**VAKAVQA	NPKTLEQHKVELS**S**VAKAVQADDAHTV	735.49	P850S	Haus6
4	GA**R**FWTGIVAMLAG	YIGHYLAMYSEGA**R**FWTGIVAMLAGAV	829.18	P96R	Tmem176a
4	YFKEWFS**I**PANLHG	HSGAEQYFKEWFS**I**PANLHGIILPRLS	889.43	R633I	Mtmr10

LLC mutations determined to be immunogenic upon mRNA-LNP immunization. Mutated amino acids (aa) are highlighted in bold. WT, wild type; AA#, position of mutated amino acid; Mut, mutation.

Table S7. Measurements of tumor volume and tumor weight of each individual mouse in different groups.

Group	No	Tumor volume (mm^3)	Average volume	Tumor weight (g)	Average weight
PBS	1	2299.28	1326.46	2.16	1.44
	2	2485.09		2.34	
	3	458.74		0.62	
	4	911.06		0.96	
	5	1010.34		1.62	
	6	794.21		0.90	
Mal	1	282.68	271.03	0.32	0.27
	2	388.79		0.34	
	3	272.55		0.28	
	4	187.53		0.20	
	5	249.67		0.24	
	6	244.94		0.21	
Nb	1	52.45	77.11	0.05	0.09
	2	81.93		0.09	
	3	72.07		0.06	
	4	76.55		0.09	
	5	87.60		0.12	
	6	92.08		0.13	

Table S8. Average percentage of different subsets of neoepitope-specific T cells.

Site	Cell type		Group (%mean)		
			PBS	Mal-LNP	Nb-LNP
Spleen	$CD8^+$	IFN-γ^+	0.75%	5.30%	16.53%
		TNF-α^+	0.22%	0.70%	7.27%
	$CD4^+$	IFN-γ^+	0.68%	1.09%	6.77%
		TNF-α^+	0.13%	0.11%	22.38%
Lymph node	$CD8^+$	IFN-γ^+	0.82%	5.34%	16.54%
		TNF-α^+	0.09%	0.44%	7.54%

$CD4^+$	IFN-γ^+	0.67%	1.04%	7.33%
	TNF-α^+	0.07%	0.10%	24.45%

Table S9. Quantification of neoantigen-specific T cell responses by ELISpot assay.

Peptide	IFN-γ spots (mean)		
	PBS	Mal-LNP	Nb-LNP
Dhrs9	6.4	287.0	551.0
Pank3	6.4	118.0	386.0
Xpo4	4.2	236.3	439.7
Gins4	0.2	190.7	423.0
Dyrk3	1.4	203.0	564.0
Mastl	2.6	29.0	164.7
Sec22c	3.0	35.3	128.0
Mmp3	4.4	18.3	52.0
Mybl1	1.8	32.3	65.0
Mtmr10	4.0	22.7	124.0
